# Homework Responsibility and Parent–Adolescent Communication: Maternal and Paternal Associations with Academic and Psychological Adjustment Among Chinese Adolescents

**DOI:** 10.3390/bs16101898

**Published:** 2026-10-14

**Authors:** Siqi Zhang, Ziying Mei, Shuya Yan

**Affiliations:** 1 Faculty of Economics and Management, Xi’an Fanyi University, Xi’an 710105, China; zsq@xafy.edu.cn; 2 School of Ethnology and Sociology, Yunnan University, Kunming 650091, China; 3 School of Accounting and Finance, Ningxia Polytechnic University of Business and Technology, Yinchuan 750021, China; yanya1224@sina.com

**Keywords:** homework responsibility, parent–adolescent communication, cognitive ability, academic achievement, depressive symptoms

## Abstract

Responsibility for homework and parent–adolescent communication may have different associations with adolescent adjustment. Using two waves of the China Education Panel Survey (2013–2015), we examined these parent-specific behaviors in outcome-specific analytic samples ranging from 7457 to 7604 adolescents. Ordinary least squares models included the four parental measures jointly and adjusted for corresponding baseline outcomes, covariates, and class fixed effects. The principal predictors and outcomes were measured concurrently at follow-up. Maternal communication was positively associated with cognitive ability and academic achievement, whereas maternal homework responsibility had negative associations in the additive main models. Communication with either parent and paternal responsibility were associated with fewer depressive symptoms. Direct coefficient comparisons distinguished maternal responsibility from maternal communication across all three outcomes after multiple-testing adjustment. These associations were small and do not establish causal effects. The study adds a joint, parent-specific comparison across academic and psychological outcomes, distinguishing reported homework responsibility from communication frequency rather than treating involvement as a single behavior.

## 1. Introduction

Parents’ involvement in education remains relevant as children enter adolescence, but what it entails changes as students assume more responsibility for their own learning. Helping with an assignment, discussing a difficulty at school, and expressing educational expectations all count as involvement, yet they need not have the same relationship with development. Reviews generally report positive associations between parental involvement and achievement, engagement, motivation, and social or emotional adjustment, while documenting substantial variation across activities (Barger et al., 2019; Boonk et al., 2018). S. Kim’s (2022) second-order synthesis of 23 meta-analyses and 1177 primary studies estimated average associations of 0.18 in observational research and 0.16 in intervention research. Expectations and aspirations had the strongest associations, whereas findings for homework involvement were mixed. Barger et al. (2019), drawing on 448 independent studies, likewise found small positive associations for most forms of naturally occurring involvement but a negative association between homework assistance and achievement. Longitudinal research also distinguishes the academic and psychological associations of school-based involvement, home-based involvement, and academic socialization (M.-T. Wang & Sheikh-Khalil, 2014). The activity being measured is therefore central to interpreting a parental-involvement association.

The distinction between task-oriented and relational involvement helps organize this comparison. Grolnick and Slowiaczek (1994) described involvement as multidimensional, distinguishing behavioral, cognitive-intellectual, and personal aspects. Pomerantz et al. (2007) further emphasized how involvement is provided, toward whom, and for what purpose. Self-determination theory directs attention to whether interactions support autonomy, competence, and relatedness, rather than simply how much parents participate (Ryan & Deci, 2000). The multidimensional framework motivates separating responsibility for schoolwork from recurrent conversation. Homework responsibility identifies the parent reported to be directly responsible for supporting homework and study. Parent–adolescent communication records how often conversations cover school experiences, relationships, and personal concerns. Responsibility concerns an educational task in which guidance may support competence, while controlling assistance may restrict autonomy. Communication provides occasions to express concerns and experience relatedness, but frequent conversations need not be supportive. These are possible theoretical links, not mechanisms measured by the CEPS items. We treat the indicators as observable aspects of involvement, not as measures of autonomy support, need satisfaction, or two complete parenting styles. Their boundaries overlap: homework support may include emotional reassurance, and conversations may concern school tasks. The analysis therefore compares reported behaviors without treating communication frequency as relationship quality or homework responsibility as controlling involvement. Following 463 adolescents from seventh through eleventh grade, Hill et al. (2004) found that earlier parental academic involvement was associated with later behavior and educational aspirations, with academic associations varying across family and demographic groups. This wider developmental perspective supports examining cognition, examination performance, and depressive symptoms together.

Homework research shows why direct responsibility for schoolwork should not automatically be read as an educational advantage. Hill and Tyson’s (2009) meta-analysis of middle-school studies found that academic socialization had a more consistently favorable association with achievement than direct homework help. Patall et al. (2008), synthesizing 20 correlational studies, reported positive average associations for elementary and high school students but a negative association for middle-school students; results also differed between mathematics and verbal outcomes. A recent three-level meta-analysis found a small negative overall association between parental homework involvement and achievement, with more favorable findings for autonomy-supportive involvement and weaker or inconsistent findings for several other forms of help (J. Xu et al., 2024). That synthesis also reported a negative average association for maternal involvement but no clear average association for paternal involvement. A mathematics-specific meta-analysis distinguished positive associations for supportive involvement from negative associations for intrusive involvement (Jiang et al., 2023). Studies of specific forms of homework support help explain this variation. Dumont et al. (2014) examined the quality of homework involvement and its reciprocal relations with academic functioning, while J. Xu et al. (2018) distinguished autonomy-oriented from content-oriented support in a two-wave study of ninth graders. Moroni et al. (2015), using data from 1685 Swiss students in Grades 5 and 6, found that help frequency and perceived intrusiveness were negatively associated with sixth-grade achievement, whereas perceived supportiveness was positively associated with it after prior achievement was considered. In a nine-month study of 370 Chinese mothers and fourth graders, Shi et al. (2024) linked constructive involvement to better academic and emotional functioning and unconstructive involvement to poorer academic functioning, mainly when children showed helplessness during homework. Their academic measures concerned motivation and self-efficacy rather than examination scores. Although the sample was younger than ours, the findings show why responsibility alone cannot identify the quality of assistance. Frequency, responsibility, and quality therefore describe different aspects of homework involvement. Parents may also increase assistance when children encounter difficulties, making a negative association compatible with a response to poor performance rather than a harmful consequence of help (Silinskas et al., 2013; Yang, 2025). Information about earlier academic functioning is particularly relevant when interpreting current responsibility for schoolwork.

Parent–adolescent communication provides a comparison with direct responsibility for schoolwork. As adolescents spend more time outside the home, conversations can give parents information about school experiences and give adolescents opportunities to discuss concerns. Research on academic socialization and parent–child discussion links these activities to engagement and achievement, as well as emotional functioning (Cheung & Pomerantz, 2011; M.-T. Wang & Sheikh-Khalil, 2014). Interpreting these findings requires attention to how communication was measured. Zapf et al. (2024) reviewed 37 reports representing 26 studies and found associations between adolescent-rated communication quality and several mental health outcomes, although most studies were cross-sectional. Tang et al.’s (2020) synthesis of 164 studies of secondary-school students in mainland China identified poor parent–child communication as a relatively strong correlate of depressive symptoms. These findings concern broader or quality-based assessments and do not establish that frequency alone is beneficial. Trager et al. (2025), following mother–adolescent and father–adolescent dyads in the Netherlands for one year, also found that digital communication content, rather than overall frequency, differentiated associations with depressive and anxiety symptoms. This channel-specific evidence reinforces the distinction between how often families communicate and what their exchanges involve. Studies using the China Education Panel Survey (CEPS) measure communication frequency more directly. Zhang et al. (2021a) found that more frequent communication with fathers and mothers was associated with fewer depressive symptoms, with some variation by age and gender. R. Wang et al. (2022) examined school-life experience, learning difficulties, and confidence in the future as possible pathways; their mediation analyses used eighth-grade cross-sectional data and did not establish temporal ordering. K. Liu (2023) considered mental unwellness and subject-specific academic performance together, distinguishing father–adolescent and mother–adolescent communication. These studies support examining communication across several outcomes while keeping frequency separate from the openness, supportiveness, or content of an exchange.

Separating mothers and fathers is equally important for interpreting these behaviors. More frequent reported involvement by one parent does not necessarily imply a stronger association with adolescent outcomes. S. W. Kim and Hill’s (2015) meta-analysis found broadly similar academic associations for maternal and paternal involvement overall, together with variation across particular activities. Jeynes (2015) also reported a positive average association between father involvement and educational outcomes in a synthesis of 66 studies. Evidence from Taiwan, however, indicated greater predictive value for maternal involvement across several parent-specific measures (Hsu et al., 2011). In mainland China, S. Liu et al. (2023) linked both maternal and paternal emotional involvement to academic engagement, while associations for cognitively oriented involvement varied by developmental stage. Parent-specific comparisons also matter for psychological adjustment. Zhang et al. (2022), for example, examined father–adolescent and mother–adolescent relationships separately in relation to later depressive symptoms, although those relationship measures did not describe responsibility for homework. These findings offer no simple basis for treating one parent as the primary source of developmental support. Comparisons need to specify the behavior and the outcome. Because parents’ activities may be correlated within a family, including their measures in separate regressions does not provide the same comparison as estimating them jointly. Direct tests of coefficient differences are needed to evaluate these comparisons: statistical significance for one parent and nonsignificance for the other do not establish a difference between parents.

Chinese research already addresses several parts of this comparison. Cheung and Pomerantz (2011) found that parental involvement was more closely connected to control and less closely connected to autonomy support in China than in the United States, even though its academic associations were similar across the two settings. S. W. Kim’s (2020) meta-analysis of 15 East Asian studies found the strongest achievement association for academic socialization, followed by home-based and school-based involvement. More recently, Cui and Liu (2025) synthesized 363 regional samples comprising 399,375 Chinese students. Broad involvement in learning and daily life had small positive achievement associations, which were stronger in Year 4 than Year 8. These broader domains are not equivalent to homework responsibility, so their positive associations do not imply that every specific activity is similarly related to achievement. CEPS studies have examined home-based involvement and academic socialization in relation to school performance and behavior (Duan et al., 2018), and cognitive ability as a possible pathway linking involvement to academic performance (Y. Li et al., 2019). Using CFPS data, Kang et al. (2024) considered pooled parental involvement, depressive symptoms, and reported academic performance. More recent studies have examined reciprocal involvement-achievement associations (Yang, 2025), parent-specific relationships and academic performance (Qin et al., 2025), and communication, shared activities, and closeness alongside cognitive and non-cognitive development (H. Wang et al., 2026). Two studies are especially close to our question. Q. Xu and Wang (2019) analyzed maternal and paternal involvement in separate models, distinguishing transactional activities, communication, and relationship closeness across adolescent outcomes. J. Li et al. (2024) examined homework involvement, parent–child communication, and negative emotions among Chinese students. The remaining question is how these specific responsibility and communication measures compare within and between parents when estimated jointly across academic and psychological outcomes, with adjustment for corresponding baseline functioning.

We use the 2013–2014 and 2014–2015 CEPS waves to compare these parent-specific measures. The four measures are paternal homework responsibility, maternal homework responsibility, father–adolescent communication frequency, and mother–adolescent communication frequency. Responsibility is reported in the parent questionnaire, communication in the student questionnaire, and outcomes come from cognitive assessments, school examination records, and adolescent reports of depressive symptoms. Cognitive ability and examination performance are considered separately because a reasoning assessment and relative performance in school subjects need not capture the same aspect of academic functioning. All four parental measures enter the same model, alongside adolescent and family covariates, follow-up class fixed effects, and the corresponding baseline outcome. Each association is thus estimated conditional on the other measured parental behaviors, prior adjustment, and observed context. Because the principal exposures and outcomes were measured at the same follow-up wave, these estimates do not identify the consequences of changing parenting practices. The use of several reporters provides distinct sources of information, but it also means that behavioral domain and reporter differ together. We therefore interpret the comparison as one between these particular measures rather than attributing every difference to task-oriented versus relational involvement. Formal coefficient contrasts quantify the differences and their uncertainty without relying on separate significance tests.

The primary analyses address two theory-informed research questions. First, how are each parent’s homework responsibility and communication frequency associated with cognitive ability, academic achievement, and depressive symptoms when all four measures are included together? Second, how large are the father–mother differences within each behavior and the responsibility–communication differences within each parent? We frame these as research questions rather than prespecified directional hypotheses: existing evidence does not imply a consistent ordering of parents or behaviors across outcomes. A secondary question asks whether the associations differ by student sex. Exploratory analyses examine the four homework-responsibility configurations and whether the responsibility–communication contrast differs between mothers and fathers. These extensions qualify interpretation but are not the basis for the primary conclusions.

## 2. Materials and Methods

We linked two waves of the China Education Panel Survey (CEPS) to examine the associations of homework responsibility and parent–adolescent communication with adolescent adjustment. We specified the measures and models separately for cognitive ability, academic achievement, and depressive symptoms, with the same four parent-specific predictors included in each model.

### 2.1. Data Source and Analytic Sample

The CEPS is a school-based longitudinal survey directed by Weidong Wang and administered by the National Survey Research Center at Renmin University of China. The 2013–2014 baseline survey used a stratified, multistage probability-proportional-to-size sampling design. After selecting 28 county-level units, the survey sampled four junior-high schools in each county or district and up to two seventh-grade and two ninth-grade classes per school. All classes were included when a grade had only one or two classes; otherwise, two were randomly selected. All students in the sampled classes were invited to participate. The baseline sample comprised 19,487 students in 438 classes and 112 schools, including 10,279 seventh-grade students and 9208 ninth-grade students (China Education Panel Survey Project Team, 2015). Students, parents or guardians, teachers, and school administrators provided questionnaire information. The survey also administered cognitive assessments and obtained examination records from schools. A published CEPS analysis reports that Renmin University of China approved the original data collection and that adolescents and their parents provided written informed consent (Duan et al., 2018).

We followed the baseline seventh-grade cohort into the 2014–2015 survey, when most students were in eighth grade. CEPS successfully followed 9449 of the 10,279 original cohort members, a follow-up rate of 91.9%. The released Wave 2 student file contains 10,750 records: the 9449 followed students, 830 records retained for original students who were not successfully followed, and 471 newly sampled students (China Education Panel Survey Project Team, 2017). Newly sampled students were ineligible for our models because they did not have a corresponding baseline outcome. We linked the Wave 1 student file, Wave 2 student file, and Wave 2 parent file one-to-one using the deidentified student identifier provided by CEPS. Each observation represented one adolescent.

Among the 9449 successfully followed adolescents, 300 had missing homework-responsibility information. Requiring both communication scores excluded a further 462, leaving 8687 adolescents with all four parental measures. For each outcome, we then required complete information on the follow-up outcome, its baseline counterpart, all model covariates, follow-up class and school identifiers, and the CEPS longitudinal sampling weight. The weight requirement made the unweighted primary and weighted sensitivity samples comparable. Final samples included 7604 students for cognitive ability, 7457 for academic achievement, and 7477 for depressive symptoms. Appendix A Table A1 reports sequential exclusions and variable-specific missingness; these variable-specific counts overlap and should not be summed. Baseline comparisons showed that included adolescents had higher cognitive and achievement scores and fewer depressive symptoms than followed adolescents excluded from each model (Appendix A Table A2). All three samples included students from 112 schools, with 295, 290, and 295 follow-up classes, respectively. Descriptive statistics and correlations used the common sample of 7245 adolescents with complete data for all three outcomes. Of these, 5918 lived with both biological parents. Regression models used the larger outcome-specific samples. Limited item nonresponse was permitted when calculating the communication and symptom scores, as described below; after scoring, records missing any required model variable were excluded. We did not impute missing responses. Complete-case analysis can be sensitive to why information is missing; having complete records does not by itself rule out outcome-related selection (White & Carlin, 2010).

The data were obtained from the CEPS public-use releases distributed through the Chinese National Survey Data Archive. Access to these releases is subject to the archive’s registration and data-use requirements and does not permit unrestricted redistribution. This study analyzed deidentified secondary records and involved no new data collection or contact with participants.

### 2.2. Measures

#### 2.2.1. Homework Responsibility

Homework responsibility was measured using Question A15 of the Wave 2 parent questionnaire. The item asked which family members were usually directly responsible for supporting the child’s homework and studies. Respondents could select more than one option: no one, father, mother, siblings, grandparents, or other people. We constructed separate father and mother indicators, coded 1 when that parent was selected and 0 when the parent was not selected. Missing responses remained missing. The item did not require respondents to select a single person as primarily responsible, and the two parental indicators were not mutually exclusive. Both indicators equaled 1 when both parents were selected, and both equaled 0 when neither parent was selected. A 0 indicates that the parent was not identified as directly responsible; it does not establish that the parent never helped. Other family members could be selected in any of the four parental configurations.

These indicators describe reported responsibility for an activity, rather than the amount, quality, or instructional style of the help provided. They do not distinguish supportive explanations from monitoring, pressure, or intrusive assistance. We used this item to examine responsibility separately for mothers and fathers while allowing both parents to be identified. It is not a measure of overall parental involvement or a validated scale of task-oriented parenting. We included both indicators in the main models and standardized them for comparisons with communication frequency. Sensitivity analyses used their original 0/1 coding or substituted reported average daily homework-support hours. In constructing the hours measure, we assigned 0 when the parent was not selected as directly responsible. For selected parents, reports between 0 and 8 h per day were retained; values outside this analysis-defined range were treated as missing. This assigned zero reflects the response to the responsibility item; it is not separate evidence that an unselected parent provided no help. Internal-consistency coefficients are not applicable to these single-item indicators and time reports.

#### 2.2.2. Parent–Adolescent Communication Frequency

Communication frequency was measured from adolescents’ Wave 2 reports of conversations with their father and mother about four topics: events at school, relationships with classmates, relationships with teachers, and personal concerns or worries. These CEPS items have also been used in research on communication and depressive symptoms (R. Wang et al., 2022). Each response was coded 1 (never), 2 (sometimes), or 3 (often). We calculated separate father–adolescent and mother–adolescent communication scores as the mean of the four items, requiring at least three valid responses for each parent. Higher scores indicate more frequent communication across the listed topics. Cronbach’s alpha was 0.82 for father–adolescent communication and 0.84 for mother–adolescent communication. The scores do not measure whether conversations were warm, open, supportive, or initiated by the parent or adolescent.

The baseline communication scores used in the temporal-ordering sensitivity analysis covered five Wave 1 topics: school events, relationships with friends, relationships with teachers, the adolescent’s mood, and personal concerns or worries. The response categories were the same as at follow-up. We required at least four valid items to calculate each parent-specific mean. Baseline alpha coefficients were 0.83 for father–adolescent communication and 0.81 for mother–adolescent communication. Because the topic sets differed between waves, the baseline and follow-up scores were not treated as an invariant repeated scale.

All alpha coefficients reported here were calculated among adolescents in the common three-outcome sample who answered every item of the relevant scale (Cronbach, 1951). Alpha describes internal consistency; it does not establish construct validity or unidimensionality (Sijtsma, 2009). Supplementary single-factor analyses examined the communication and symptom item sets at each wave (Section 2.3). Measurement invariance was not tested. We therefore analyzed homework responsibility and communication frequency as observed behaviors, not as validated scales of task-oriented and relational parenting.

#### 2.2.3. Cognitive Ability

Cognitive ability was measured using the CEPS standardized assessment. The follow-up assessment contained 35 items and had a 30 min time limit. Its language, graphical and spatial reasoning, calculation, and logical problem-solving tasks assessed reasoning beyond knowledge of a particular school subject. CEPS assigned one of three test forms according to the student’s baseline cognitive score to improve discrimination across ability levels. We used the follow-up score estimated by CEPS with a three-parameter item response theory model and standardized it across valid observations in the linked baseline cohort. The corresponding CEPS-provided baseline score was standardized separately and entered as the prior cognitive outcome (China Education Panel Survey Project Team, 2015, 2017). We analyzed the released aggregate scores rather than rescoring the test items. We therefore did not re-estimate an internal-consistency coefficient from the aggregate scores.

#### 2.2.4. Academic Achievement

Academic achievement was measured from Chinese, mathematics, and English scores on the autumn 2014 midterm examinations, obtained directly from participating schools (China Education Panel Survey Project Team, 2017). We divided each nonnegative subject score by its reported maximum when the maximum was positive. Ratios were retained as supplied and were not capped at 1. Each subject ratio was standardized within the student’s follow-up class using all available valid scores before analytic-sample exclusions. We averaged the three class-standardized subject scores, requiring all three subjects, and standardized the composite across the linked baseline cohort. This composite was the primary academic-achievement outcome. It measures relative examination performance within classes, not performance on a nationally equated test. Class standardization does not establish that schools used equivalent examination content or difficulty.

For the baseline achievement covariate, we used the CEPS-provided Chinese, mathematics, and English scores from the autumn 2013 midterm examinations. CEPS standardized those subject scores within school and grade to a mean of 70 and a standard deviation of 10 (China Education Panel Survey Project Team, 2015). We required all three baseline subjects, averaged them, and standardized the resulting composite. Subject-specific sensitivity models adjusted for the corresponding baseline subject score. We also examined an alternative outcome: the standardized mean of the three follow-up score-to-maximum ratios, again requiring all three subjects. School examination records were treated as performance measures, rather than as interchangeable items on a psychological scale; Cronbach’s alpha was not used to establish their validity.

#### 2.2.5. Depressive Symptoms

Depressive symptoms were assessed with the first six items of Question C25 in the follow-up student questionnaire (China Education Panel Survey Project Team, 2017). Students reported how often, during the preceding seven days, they had felt depressed, had been too low to concentrate, had felt unhappy, had felt that life was meaningless, had lacked energy to do things, or had felt sad. Responses ranged from 1 (never) to 5 (always), with seldom, sometimes, and often as the intervening categories. We calculated the mean when at least four items were answered. Higher values indicate more frequent symptoms. Cronbach’s alpha was 0.92, and the mean score was standardized across the linked baseline cohort for regression analysis. This CEPS symptom index was not a diagnostic assessment of depression, and no clinical cutoff was applied.

The baseline symptom covariate comprised five depressive-affect items covering feeling down, depressed, unhappy, that life was meaningless, and sad during the preceding seven days (China Education Panel Survey Project Team, 2015). These items used the same five frequency categories. We required at least three valid responses to calculate the mean, then standardized the index. Its alpha was 0.84. The baseline and follow-up indices describe closely related experiences, but their item sets are not identical. Baseline symptoms were therefore treated as a prior outcome covariate, not as an exactly equivalent earlier administration of the follow-up scale.

#### 2.2.6. Covariates

Adolescent covariates included sex, age, only-child status, hukou, and boarding status. Sex refers to the male/female classification recorded in CEPS from the baseline student questionnaire (item A1). It was coded 1 for girls and 0 for boys. Age was calculated as 2014 minus baseline-reported birth year, approximating age at the start of the follow-up academic year; values outside 11–18 years were treated as missing. Follow-up only-child status was coded 1 for an only child and 0 otherwise. Boarding status indicated whether the student stayed at school on Monday through Thursday nights (1 = yes, 0 = no). Hukou was coded 1 for nonagricultural or unified resident registration and 0 for agricultural registration or no registered hukou, using the four response categories in the follow-up questionnaire.

Family covariates were measured at follow-up. Students rated current family economic conditions from 1 (very difficult) to 5 (very affluent), with somewhat difficult, average, and somewhat affluent as the intervening categories. The parent questionnaire recorded each parent’s highest educational attainment separately: 1 = no schooling, 2 = primary school, 3 = junior secondary school, 4 = secondary vocational or technical school, 5 = vocational high school, 6 = general high school, 7 = junior college, 8 = bachelor’s degree, and 9 = postgraduate education or above. Economic conditions and each parent’s education were entered as numeric ordered covariates; the education codes were not converted to years of schooling. This provides a parsimonious adjustment for socioeconomic background but assumes the same incremental outcome association between adjacent categories. This restriction may be particularly strong for educational categories, which represent different qualifications rather than equal intervals of schooling. Two additional indicators recorded whether the adolescent currently lived with the biological father and biological mother, respectively (1 = selected in the household-members question, 0 = not selected). Missing covariate responses remained missing. Each fully adjusted outcome model also included the corresponding standardized baseline outcome.

### 2.3. Statistical Analysis

We calculated descriptive statistics for the common three-outcome sample and Pearson correlations among the four parental measures and three outcomes. Before outcome-specific complete-case selection, each focal predictor, outcome, baseline outcome, and alternative predictor was standardized using the mean and standard deviation of its valid observations in the linked baseline cohort. Standardization used the population-form standard deviation, with the valid-observation count in the variance denominator. A standardized variable therefore need not have an exact mean of 0 or standard deviation of 1 in a smaller analytic sample. For the binary responsibility indicators, a main-model coefficient describes a one-standard-deviation difference in the predictor, not the full difference between 0 and 1. Models using the original coding provide that binary contrast and the association per one-point difference in communication frequency. Other covariates retained the coding described above.

We compared baseline characteristics between included and excluded adolescents separately for each outcome among the 9449 successfully followed students. We also compared followed and non-followed adolescents in the original 10,279-person cohort. Comparisons were unweighted and used available observations for each variable. Signed standardized mean differences (SMDs) were group mean or proportion differences (included minus excluded, or followed minus non-followed), divided by the square root of the average group variances. Two-sided *p* values came from OLS models with only a group indicator and intercept, using school-clustered CR1 standard errors (112 schools; 111 degrees of freedom). These descriptive tests were not adjusted for multiple comparisons.

The primary analyses comprise the joint parental associations and the direct father–mother and within-parent responsibility–communication contrasts for the three outcomes. Sex interactions are secondary analyses. Weighting, sample-definition, coding, and alternative-measurement models assess sensitivity; subject-specific and baseline-communication models address supplementary questions. Homework configurations and second-order contrasts are exploratory. This hierarchy reflects the research questions rather than statistical significance. Ordinary least squares models were estimated separately for cognitive ability, academic achievement, and depressive symptoms. The fully adjusted specification was

(1) $$y_{i2}\;=\;\alpha \;+\;\beta_{1}h_{Fi}\;+\;\beta_{2}h_{Mi}\;+\;\beta_{3}c_{Fi}\;+\;\beta_{4}c_{Mi}\;+\;\rho y_{i1}\;+\;x_{i}’\gamma \;+\;\lambda_{j(i)}\;+\;\varepsilon_{i}.$$

where $y_{i2}$ is the standardized follow-up outcome for adolescent i, and $y_{i1}$ is the corresponding standardized baseline outcome. The lowercase letters h and c denote homework responsibility and communication frequency; F and M identify fathers and mothers. The four standardized follow-up predictors are therefore $h_{Fi}$, $h_{Mi}$, $c_{Fi}$, and $c_{Mi}$. The vector $x_{i}$ contains the adolescent and family covariates, $\lambda_{j(i)}$ denotes the follow-up class fixed effect, and $\varepsilon_{i}$ is the error term. The intercept is α; $\beta_{1}$ through $\beta_{4}$ are the parental-measure coefficients, ρ is the baseline-outcome coefficient, and γ contains the covariate coefficients. A separate set of coefficients was estimated for each outcome. Each parental coefficient thus describes an association conditional on the other three parental measures, the covariates, class membership, and prior adjustment in the same outcome domain.

We estimated five nested specifications on a fixed outcome-specific complete-case sample. Model 1 contained the four parental measures; Model 2 added adolescent covariates; and Model 3 added family covariates. Model 4 included follow-up class fixed effects. For academic achievement, within-class standardization expresses performance relative to classmates, whereas class fixed effects restrict regression comparisons to adolescents within the same class. Standardizing the outcome alone does not remove between-class variation in parental measures or covariates. Model 5 added the corresponding baseline outcome and was the primary specification in Equation (1). Holding the sample fixed ensured that coefficient changes across these models were not due to changes in sample composition. The principal parental predictors and outcomes were measured concurrently at follow-up. Model 5 therefore estimates follow-up associations conditional on the corresponding baseline outcome, covariates, and class fixed effects. These are between-adolescent associations, not estimates of within-person change or effects of changes in parental behavior. Baseline adjustment does not establish temporal precedence for the follow-up parental measures, remove reverse causation, or identify causal effects.

Four coefficient contrasts were specified for each outcome: father-minus-mother homework responsibility, father-minus-mother communication frequency, and responsibility-minus-communication within each parent. Homework responsibility is a binary indicator, whereas communication is a multi-item frequency score. The contrasts compare standardized conditional associations; one-standard-deviation differences in the two measures do not represent equivalent amounts of parental behavior, time, or effort. We calculated each contrast from the joint coefficient covariance; differences were not inferred from separate significance tests (Gelman & Stern, 2006). We report the contrast estimates, 95% confidence intervals, unadjusted *p* values, and Benjamini–Hochberg-adjusted *p* values across the 12 contrasts. During manuscript revision, an additional exploratory contrast compared the maternal responsibility–communication difference with the corresponding paternal difference for each outcome; these three tests were adjusted as a separate family. They were not preregistered. The notation *q* denotes the Benjamini–Hochberg-adjusted *p* value reported alongside the unadjusted *p* value (Benjamini & Hochberg, 1995).

Standard errors used the CR1 finite-sample correction and were clustered at the school level to allow residual dependence within schools (Cameron & Miller, 2015). The three main samples each contained 112 school clusters. Coefficient and contrast tests were two-sided and used a t distribution with 111 degrees of freedom; pointwise 95% confidence intervals used the same covariance matrix and critical values; the intervals were not adjusted for multiple testing. The principal significance threshold was 0.05. Tables use *, **, and *** for unadjusted *p* < 0.05, *p* < 0.01, and *p* < 0.001, respectively. No significance symbols are assigned to *p* ≥ 0.05.

We examined sex differences in pooled extensions of the fully adjusted model. Each parental predictor was interacted with the indicator for girls, retaining all four main effects, the covariates, class fixed effects, and baseline outcome. Boys were the reference group. Girls’ coefficients were calculated as the sum of the focal and interaction coefficients, with standard errors obtained from their joint covariance. Each interaction directly tested the corresponding sex difference; inference was based on these tests, not on separate within-sex significance tests.

Sensitivity analyses examined weighting, sample definition, and measurement. Primary models were unweighted and described conditional associations in the observed longitudinal sample, not nationally representative population estimates. We also estimated the fully adjusted models using the CEPS Wave 1–Wave 2 longitudinal weight, since weighting can change regression estimates as well as descriptive statistics (Solon et al., 2015). Further analyses used the common three-outcome sample, restricted the sample to adolescents living with both biological parents, retained the original parental-measure coding, or substituted standardized homework-support hours. The temporal-ordering analysis entered both standardized baseline communication scores together, with the covariates, follow-up class fixed effects, and corresponding baseline outcome; follow-up homework responsibility was not included in that specification. Unlike the concurrent main models, this analysis relates baseline communication to later outcomes conditional on baseline functioning. The five-item baseline and four-item follow-up scores cover different topics, as described in Section 2.2.2, and each specification uses its own complete-case sample. Temporal ordering does not remove unmeasured confounding or establish causality. For academic achievement, we also examined the mean score-to-maximum ratio and the separate subjects. Alternative-measure models used complete cases for their own required variables. Benjamini–Hochberg adjustment was applied to the 12 parental coefficients from the three fully adjusted main models as a family separate from the coefficient contrasts.

To assess sensitivity to socioeconomic covariate coding, we re-estimated the three fully adjusted models with separate sets of category indicators for family economic conditions (five categories) and each parent’s educational attainment (nine categories each). The reference categories were very difficult economic conditions and no schooling; no categories were combined. Samples, standardization, other covariates, baseline adjustment, class fixed effects, and school-clustered inference were unchanged. Within each specification, Benjamini–Hochberg adjustment was applied separately to the 12 focal coefficients and the 12 coefficient contrasts.

To assess the functional form of communication, we fitted three-knot restricted cubic splines for paternal and maternal communication jointly in each fully adjusted outcome model. Knots were placed at each parent’s 10th, 50th, and 90th sample percentiles (fathers: 1, 2, and 2.75; mothers: 1.5, 2.25, and 3). The original samples, standardization, homework-responsibility indicators, covariates, baseline outcomes, class fixed effects, and school-clustered inference were retained. Parent-specific Wald tests evaluated the additional nonlinear terms, with Benjamini–Hochberg adjustment across the six tests.

We used the homework-configuration analysis to examine departures from additivity in the two responsibility indicators. We formed four mutually exclusive categories: neither parent identified, father only identified, mother only identified, and both parents identified. “Identified” denotes selection as directly responsible for homework and study; “only” distinguishes the two parents and does not exclude other helpers. Models used neither parent identified as the reference and retained both communication measures, all covariates, class fixed effects, and the corresponding baseline outcome. Linear contrasts compared the three identified-parent configurations and tested whether the both-parents coefficient departed from the sum of the father-only and mother-only coefficients. Configuration indicators were not standardized, so these estimates express outcome-standard-deviation differences between categories. The configuration analyses were exploratory. Their original *p* values are reported together with a sensitivity adjustment across all 21 configuration tests: nine category-versus-reference coefficients and 12 contrasts across the three outcomes.

As a supplementary measurement diagnostic, we fitted six separate single-factor models to the ordered communication and depressive-symptom items: father and mother communication and symptoms at each wave. We used mean- and variance-adjusted weighted least squares (WLSMV), with school-clustered standard errors and fit tests. Each model used members of the common three-outcome sample with complete responses to its items, matching the alpha calculation. Models were unweighted; no items were deleted and no residual correlations were added. We examined standardized loadings, scaled-test fit indices, and the additional robust fit indices returned by lavaan. These analyses assessed the single-factor representation of the observed item sets, not measurement invariance or regression sensitivity to alternative scoring.

The descriptive and regression analyses were conducted using Stata/SE 17.0. The supplementary factor analyses used R 4.6.1 with the lavaan package, version 0.7-2 (Rosseel, 2012).

## 3. Results

After the descriptive results, we present the primary outcome models and direct coefficient comparisons, followed by secondary sex-interaction analyses and supplementary analyses. Unless stated otherwise, regression estimates refer to the fully adjusted models. The coefficients express differences in the outcome, in standard-deviation units, per one-standard-deviation difference in the predictor. Unadjusted *p* values and multiplicity-adjusted *q* values are distinguished below.

### 3.1. Sample Characteristics and Parental Measures

Table 1 describes the common three-outcome sample of 7245 adolescents. Girls constituted 50.31% of the sample, the mean age was 13.51 years (*SD* = 0.68), and 30.01% were boarding students. In the outcome-specific models, 7604 adolescents contributed to the cognitive-ability analysis, 7457 to academic achievement, and 7477 to depressive symptoms. Mothers were more often identified as directly responsible for homework support than fathers. The mother indicator was coded 1 for 3005 adolescents (41.48%), compared with 2180 (30.09%) for the father indicator. These groups overlap because both parents could be selected. On the 1–3 communication scale, the mean was 2.23 for mothers (*SD* = 0.58) and 1.94 for fathers (*SD* = 0.55). The mean depressive-symptom score was 2.15 (*SD* = 0.89) on the 1–5 scale.

Appendix A Table A2 shows observed differences associated with both complete-case selection and loss to follow-up. Across the three analytic samples, included adolescents had higher baseline cognitive scores (SMDs = 0.255–0.283), higher achievement (0.323–0.371), and lower symptom scores (−0.105 to −0.093) than followed adolescents excluded from the corresponding model. Those successfully followed also had higher baseline cognition (SMD = 0.448) and achievement (0.379), and lower symptoms (−0.211), than those not followed.

Before regression adjustment, communication frequency was positively correlated with both academic outcomes and negatively correlated with depressive symptoms (Table 2). Father–adolescent and mother–adolescent communication correlated with cognitive ability at 0.106 and 0.166, respectively, and with achievement at 0.082 and 0.140. Their symptom correlations were −0.154 and −0.142. The two communication scores were moderately correlated with one another (*r* = 0.572). Homework-responsibility correlations were smaller: the father and mother indicators correlated with cognitive ability at 0.041 and 0.061, with achievement at −0.006 and −0.032, and with symptoms at −0.079 and −0.049. The zero-order correlations therefore differed from some of the patterns observed after covariate adjustment.

The single-factor models fitted the follow-up communication items more closely than the baseline items. Scaled RMSEA was 0.019 and 0.039 for father and mother communication at follow-up, compared with 0.246 and 0.223 at baseline. For depressive symptoms, scaled CFI was 0.980 at follow-up and 0.982 at baseline, whereas scaled RMSEA was 0.141 and 0.079, respectively. The additional robust indices also indicated poor fit for baseline communication and remaining misfit for symptoms. Thus, the diagnostics did not support a unidimensional interpretation of all six item sets.

### 3.2. Homework Responsibility, Communication, and Cognitive Ability

Mother–adolescent communication was positively associated with cognitive ability in all five specifications (Table 3). Its coefficient declined from 0.157 in Model 1, which contained only the four parental measures, to 0.050 in the fully adjusted model (*SE* = 0.011, *p* < 0.001). Maternal homework responsibility followed a different pattern. Its coefficient changed from 0.024 in Model 1 (*p* = 0.115) to −0.020 in Model 2 (*p* = 0.108), when adolescent covariates were added; neither estimate met the 0.05 threshold. This was the largest change between successive models. The coefficient was −0.043 after family covariates were added in Model 3 and −0.048 with class fixed effects in Model 4. Adding baseline cognition attenuated the estimate to −0.036 in Model 5 (*SE* = 0.010, *p* < 0.001), rather than producing the sign change. After adjustment, the maternal associations differed in direction as well as magnitude.

In the fully adjusted model, the two paternal coefficients were close to zero. Paternal homework responsibility rounded to 0.000 (95% CI [−0.021, 0.021], *p* = 0.997), and father–adolescent communication had a coefficient of −0.008 (95% CI [−0.029, 0.014], *p* = 0.499). Panel B reports the unadjusted *p* values and BH-adjusted *q* values for the Model 5 coefficient comparisons. The father–mother communication difference remained significant after adjustment across the 12 contrasts (*p* = 0.003, *q* = 0.013), whereas the responsibility difference did not (*p* = 0.033, *q* = 0.056). Within mothers, the responsibility-minus-communication difference was −0.085 (95% CI [−0.115, −0.056], *q* < 0.001). The contrast concerns standardized conditional associations between a binary responsibility indicator and a communication frequency score, not equivalent amounts of parental involvement. The corresponding paternal difference was not significant (*p* = 0.654, *q* = 0.714).

### 3.3. Homework Responsibility, Communication, and Academic Achievement

Table 4 shows a similar contrast between maternal homework responsibility and communication for composite examination performance. In Model 5, maternal homework responsibility was associated with lower achievement (*β* = −0.020, *SE* = 0.008, *p* = 0.011), while mother–adolescent communication was associated with higher achievement (*β* = 0.041, *SE* = 0.008, *p* < 0.001). The maternal homework-responsibility coefficient was negative in all five models (−0.062, −0.059, −0.065, −0.065, and −0.020, respectively). Its largest change occurred between Models 4 and 5, when baseline achievement was added, rather than when adolescent or family covariates were introduced. The maternal communication coefficient also decreased after baseline adjustment, from 0.101 to 0.041.

Neither paternal coefficient met the 0.05 threshold in the fully adjusted model. The estimate was 0.005 for homework responsibility (95% CI [−0.009, 0.018], *p* = 0.516) and 0.011 for communication (95% CI [−0.004, 0.027], *p* = 0.139). The unadjusted father–mother comparisons had *p* values of 0.030 for responsibility and 0.037 for communication, but both had adjusted *q* values of 0.056. Neither father–mother comparison met the 0.05 threshold after adjustment across the 12 contrasts. The within-mother responsibility–communication difference remained significant (difference = −0.061, 95% CI [−0.086, −0.036], *q* < 0.001); the within-father difference did not (*p* = 0.551, *q* = 0.661).

### 3.4. Homework Responsibility, Communication, and Depressive Symptoms

More frequent communication with either parent was associated with fewer depressive symptoms (Table 5). The fully adjusted coefficients were −0.054 for father–adolescent communication (*SE* = 0.016, *p* < 0.001) and −0.052 for mother–adolescent communication (*SE* = 0.017, *p* = 0.003). The direct comparison did not detect a difference between these coefficients (*p* = 0.941).

**Figure 1:**
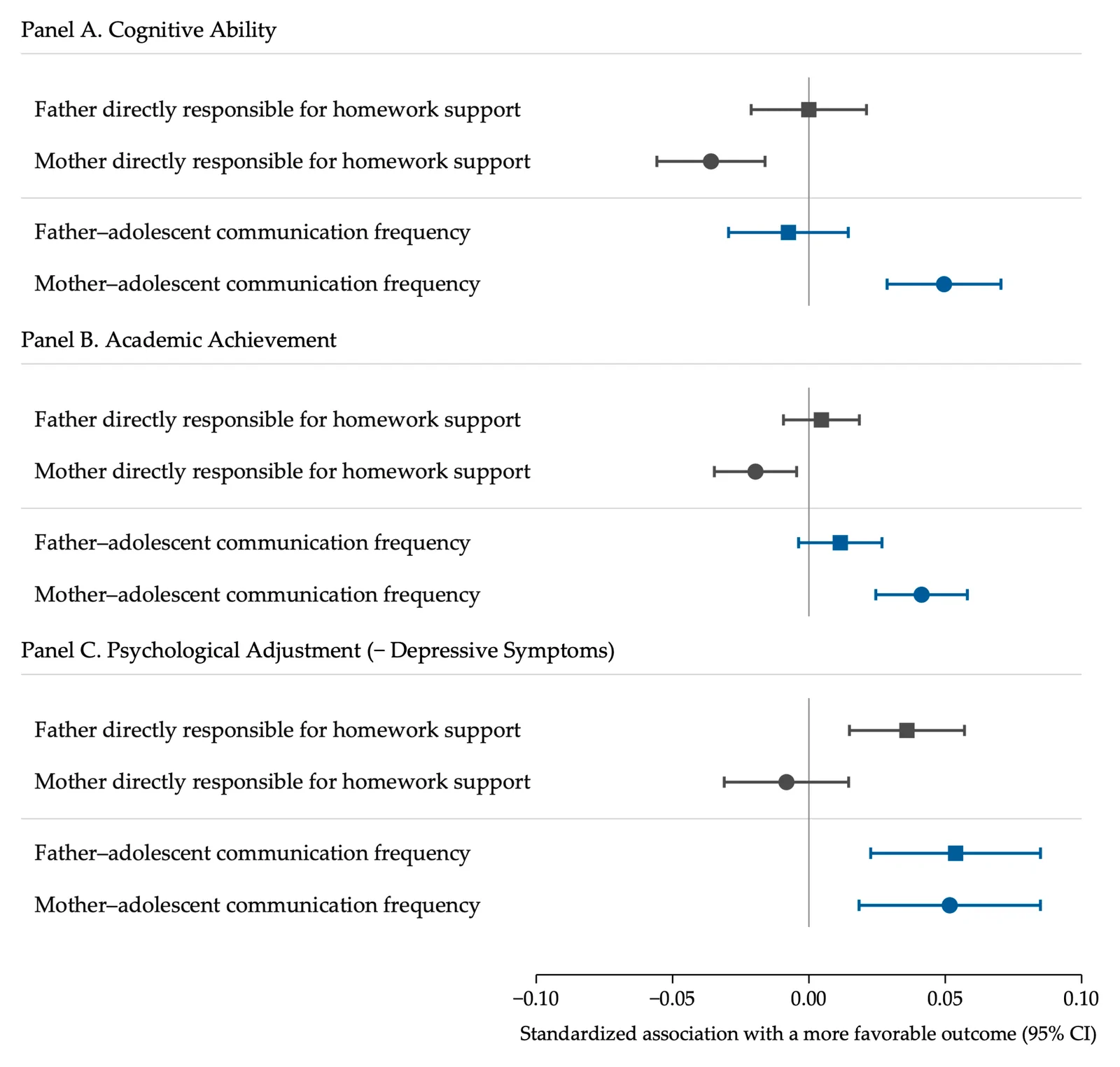
Parent-specific homework responsibility and communication: associations with academic and psychological adjustment. Notes: Points are standardized OLS coefficients from Model 5 of Table 3, Table 4 and Table 5; whiskers are 95% t confidence intervals based on CR1 standard errors clustered by school (112 schools). All models include child and family controls, Wave 2 class fixed effects, and the corresponding Wave 1 outcome. Samples are N = 7604 for Cognitive Ability, N = 7457 for Academic Achievement, and N = 7477 for Depressive Symptoms. In Panel C, coefficients for Depressive Symptoms are multiplied by −1 for display only, so positive values consistently indicate more favorable adjustment. Source: CEPS, 2013–2014 baseline and 2014–2015 follow-up.

For homework responsibility, the pattern differed between the parents. Paternal responsibility was associated with fewer symptoms (*β* = −0.036, *SE* = 0.011, *p* = 0.001), whereas maternal responsibility was not significantly associated with symptoms (*β* = 0.008, 95% CI [−0.015, 0.031], *p* = 0.475). The father-minus-mother responsibility difference was −0.044 (95% CI [−0.079, −0.010], *p* = 0.013, *q* = 0.031). Maternal responsibility also differed from maternal communication (difference = 0.060, 95% CI [0.019, 0.101], *p* = 0.004, *q* = 0.013). The corresponding paternal difference was not significant (*p* = 0.353, *q* = 0.471). Figure 1 displays the fully adjusted coefficients and 95% confidence intervals for all three outcomes. Symptom coefficients are multiplied by −1 in Panel C for display only, placing more favorable adjustment on the positive side of each panel.

### 3.5. Secondary Analyses: Sex Interactions

None of the 12 parental-measure-by-sex interactions across the three outcomes reached the 0.05 threshold (Table 6). The 12 interaction-test *p* values ranged from 0.107 to 0.965.

The sex-specific coefficients are presented descriptively, and separate within-sex significance tests do not establish sex differences. Inference about differences between boys and girls is based on the interaction tests. The nonsignificant interactions do not establish equivalence.

### 3.6. Sensitivity and Supplementary Analyses

Appendix A Table A3 shows that weighting, use of the common sample, and restriction to adolescents living with both biological parents generally preserving the main patterns. Exceptions included a negative father–adolescent communication coefficient for cognition in the weighted model and a maternal responsibility coefficient that did not meet the 0.05 threshold when achievement was measured using score-to-maximum ratios. In the separate baseline-communication models, maternal communication was positively associated with later cognition and achievement, whereas paternal communication was negatively associated with later cognition. Neither parent’s baseline communication coefficient for later depressive symptoms met the 0.05 threshold (father: *β* = −0.006, *p* = 0.693; mother: *β* = −0.029, *p* = 0.052). These are temporally ordered associations under a different measurement and model specification, not a longitudinal replication of the main models.

Categorical coding of socioeconomic covariates yielded similar focal estimates (largest absolute coefficient change = 0.0020; Appendix A Table A4). All 12 signs and significance classifications at 0.05 were unchanged, before and after Benjamini–Hochberg adjustment. Three father–mother contrasts crossed the adjusted 0.05 threshold: homework responsibility for cognition (*q* = 0.0562 to 0.0451) and achievement (*q* = 0.0562 to 0.0442), and communication for achievement (*q* = 0.0562 to 0.0483). Their directions and nominal significance were unchanged.

Spline models provided no clear evidence of nonlinearity in either parent’s communication associations. Unadjusted nonlinearity *p* values for paternal and maternal communication were, respectively, 0.403 and 0.207 for cognitive ability, 0.267 and 0.957 for academic achievement, and 0.725 and 0.238 for depressive symptoms. Benjamini–Hochberg-adjusted *q* values ranged from 0.534 to 0.957. These results did not indicate a need to replace the linear specifications, although nonsignificant tests do not establish exact linearity.

Maternal communication was positively associated with all three examination subjects (Appendix A Table A5). Maternal responsibility was negatively associated with mathematics and English, but its Chinese coefficient did not reach the 0.05 threshold. Neither paternal measure was significant for any subject. The main significance patterns were unchanged with the original predictor coding; replacing responsibility with reported support hours retained the negative maternal academic associations and negative paternal symptom association.

The exploratory homework-configuration analysis qualifies the maternal achievement finding (Appendix A Table A6). Relative to neither parent being identified as directly responsible, identification of the mother alone was associated with lower cognition and achievement; both associations survived adjustment across the 21 configuration tests (*q* = 0.021 for each). The both-parents configuration did not differ significantly from the neither-parent reference in achievement. Its comparison with the mother-only configuration and the achievement nonadditivity test were significant before adjustment but not afterward. No other configuration test met the adjusted 0.05 threshold. Thus, the configuration results do not establish that the maternal achievement association differs according to whether the father was also identified as responsible.

### 3.7. Multiple Testing in the Primary Analyses

After Benjamini–Hochberg adjustment, five of the 12 direct coefficient contrasts remained significant, while three nominally significant academic father–mother contrasts did not (Appendix A Table A7). All seven significant focal coefficients in the main models survived adjustment across the 12 main-model coefficients. The exploratory second-order comparisons are reported separately in Appendix A Table A7; they are not used to establish the primary conclusions.

## 4. Discussion

Using two waves of CEPS, this study compared maternal and paternal homework responsibility and communication frequency in relation to cognitive ability, academic achievement, and depressive symptoms. The main estimates describe concurrent follow-up associations conditional on the corresponding baseline outcome, not prospective associations of the follow-up parental measures. The within-mother responsibility–communication contrasts remained significant across all three outcomes after multiple-testing adjustment. The small, outcome-specific associations do not support treating greater involvement as uniformly favorable or ranking mothers and fathers overall.

### 4.1. Interpretation of the Findings

The maternal academic findings are consistent with research distinguishing the amount of homework help from adolescents’ experience of that help. Self-determination theory suggests asking whether homework assistance supports competence and autonomy, not merely who provides it (Ryan & Deci, 2000). J. Xu et al.’s (2024) meta-analysis reported a small negative overall association between homework involvement and achievement but a positive association for autonomy-supportive involvement. Moroni et al. (2015) found a similar distinction among 1685 Swiss students: help frequency was negatively associated with achievement, while perceived supportiveness was positively associated with it. Following mother–child dyads across Grades 6, 7, and 9, Silinskas et al. (2022) also linked direct maternal homework involvement to lower scores on several subsequent academic skills and mothers’ perceptions of adolescent autonomy to task values. Recent Chinese evidence also separates constructive from unconstructive homework involvement, with different longitudinal associations in younger children (Shi et al., 2024). Our analysis extends this comparison to reported responsibility and communication, with limited information about help quality. These associations concern reported responsibility, not the presence or absence of homework help. A parent who was not identified as directly responsible may still assist the adolescent. A mother identified as directly responsible may explain assignments, supervise completion, or respond to difficulties. The CEPS item cannot separate these activities, so the negative coefficient provides no basis for describing maternal assistance as intrusive or harmful, or for expecting better performance if mothers withdraw help.

The adjustment patterns differed between the academic outcomes. For cognition, the sign changed after adolescent covariates were added, before adjustment for family characteristics or baseline cognition. For achievement, adding baseline performance produced the largest attenuation of the negative association. These coefficient changes depend on the sequence of adjustment and do not identify the contribution of any single covariate. One possible explanation is that difficulties draw mothers into a more direct role. Among Spanish secondary-school students, lower prior achievement was associated with greater perceived parental control of homework (Núñez et al., 2017). Yang (2025) also reported reciprocal associations between involvement and academic performance during early adolescence in China, with different patterns for autonomy support and behavioral control. These studies offer a plausible context for our results, although the reasons mothers took responsibility were not observed. Responsibility and outcomes were assessed at the same follow-up, so these results cannot establish whether academic difficulties preceded mothers’ assumption of responsibility. A baseline outcome cannot capture every emerging learning difficulty or change in family life. Associations of autonomy support with achievement and psychosocial functioning suggest a direction for more detailed measurement (Vasquez et al., 2016). Since autonomy support was not assessed here, responses to difficulties and differences in assistance quality remain explanations for further study.

The configuration analysis further qualifies the maternal achievement association. Identification of the mother alone was associated with lower achievement relative to neither parent being identified; the both-parents estimate was smaller in magnitude and imprecisely estimated. The direct both-parents versus mother-only comparison and the nonadditivity test met the unadjusted 0.05 threshold, but neither survived adjustment across all 21 configuration tests. Uncertainty about nonadditivity therefore remains, and the results do not establish an achievement advantage for the both-parents group over the neither-parent reference. The meaning of joint identification also requires care. Feinberg (2003) distinguishes the division of parenting work from agreement, support, and joint family management. Those dimensions of coordinated coparenting were not measured by the CEPS responsibility item. The reference category, “neither parent identified,” does not mean that no one helps the adolescent. It may include help from other relatives or from a parent who was not identified as directly responsible. The configurations describe who was reported to take responsibility for homework, with limited information about parenting quality or the family’s wider provision of support.

Earlier Chinese studies already distinguished parent-specific activities, communication, and adolescent outcomes (Q. Xu & Wang, 2019; J. Li et al., 2024). Our contribution is to compare the specific responsibility and communication measures jointly for both parents across three outcomes, conditional on corresponding baseline functioning. This approach follows the multidimensional view of involvement (Grolnick & Slowiaczek, 1994) and the distinction between the extent and quality of participation (Pomerantz et al., 2007). The within-mother contrasts concern these measured associations, not validated task-oriented and relational parenting styles. Homework responsibility and communication overlap in daily life but differ in response scale and reporter: responsibility is parent-reported, whereas communication is adolescent-reported. The present models cannot separate behavioral differences from reporter-related influences in these contrasts. A one-standard-deviation difference in responsibility also need not represent the same parental effort as one in communication. They do not establish that relational parenting is superior to task-oriented parenting. These contrasts do not estimate what would happen if mothers replaced homework support with more frequent communication.

Maternal communication showed a more consistent positive academic pattern than paternal communication, but the evidence does not support a general ranking of parents. The father–mother communication difference for cognition survived multiplicity adjustment; the achievement difference did not. S. W. Kim and Hill (2015) found broadly similar academic associations for maternal and paternal involvement overall, with variation across particular forms. The present estimates also concern specific behaviors and leave unmeasured each parent’s wider educational contribution through expectations, resources, routines, and other support. Paternal communication deserves particular caution because its cognitive coefficient was negative in the weighted and baseline-communication models. Cultural differences alone offer an incomplete explanation. Cheung and Pomerantz (2011) found closer connections between involvement and control in China than in the United States but similar academic associations across the two settings. Understanding the present parent-specific findings would require information about what parents and adolescents discuss, who initiates conversations, and the circumstances surrounding them.

The psychological findings differed from the academic pattern. Communication with both parents was associated with fewer depressive symptoms, with no detectable father–mother difference. Communication and symptoms were reported by the same adolescent at follow-up, so their association may partly reflect shared reporting processes. The present analysis cannot quantify this contribution. This result is consistent with CEPS research on communication frequency (R. Wang et al., 2022) and broadly accords with the literature reviewed by Zapf et al. (2024). That review primarily concerns communication quality rather than frequency. Longitudinal research on digital communication likewise distinguishes content-specific associations from overall frequency (Trager et al., 2025). Neither source establishes that the CEPS frequency index measures supportive exchanges. A relatedness-based interpretation is possible: conversations may offer opportunities to discuss worries and seek help (Ryan & Deci, 2000). These mechanisms were not measured, and frequent exchanges can also be critical or controlling. Paternal homework responsibility was associated with fewer symptoms despite its small academic coefficients. Sarkadi et al.’s (2008) review of longitudinal studies likewise supports considering fathers’ engagement across several developmental outcomes, without attributing them to a particular activity. Reported responsibility could indicate a father’s availability or regular contact, although these explanations were not tested. Within the symptom model, the associations for paternal responsibility and communication were not statistically distinguishable from one another.

The baseline-communication analyses address a different temporal question from the concurrent main models. Baseline maternal communication was associated with subsequent cognition and achievement, but neither parent’s baseline communication coefficient met the principal threshold for later symptoms. The predictors precede the outcomes, but the communication items, included parental predictors, and complete-case samples differ from those in the main models. This analysis is therefore not a longitudinal replication of the concurrent estimates. The prospective findings also require caution because the baseline communication items showed poor fit to a one-factor model, providing weaker support for unidimensionality than the follow-up items. The baseline scores summarize communication frequency across the specified topics, and their associations with later outcomes should be interpreted with this measurement limitation in mind. Neither analysis establishes that more frequent conversation prevents depressive symptoms. Nonsignificance in the baseline models does not establish an absence of association or a difference from the concurrent coefficients. Adolescents’ emotional state and willingness to disclose experiences may shape reported communication. Kerr and Stattin (2000) showed that spontaneous adolescent disclosure contributed substantially to the relationship between parental knowledge and adjustment. Z. Liu et al. (2025), using three CFPS waves, found reciprocal longitudinal associations between educational involvement and depressive symptoms, while Hou et al. (2020) found that adolescent symptoms preceded deterioration in nurturant-involved parenting in a study including mothers and fathers. Hu and Zhou (2025), following 438 adolescents who completed three waves, also reported longitudinal symptom associations involving both parental responses to emotions and adolescents’ emotional communication behaviors. Their measures concern emotion-specific processes, not conversation frequency alone. Differences in measurement and timing limit direct comparison with our study. These findings nevertheless draw attention to adolescents’ participation in communication and caution against interpreting greater conversation frequency as evidence of depression prevention.

The interaction tests did not provide statistically significant evidence of sex differences in the four associations. These results do not support recommendations for different parental roles for boys and girls. Z. Liu et al. (2025) similarly found no gender differences in their longitudinal involvement-symptom associations, although their measures differed from ours. Nonsignificant tests in either study do not establish equivalence. Assessing equivalence would require a defensible smallest meaningful difference and sufficient precision to evaluate it (Lakens, 2017). Differences in within-sex significance should not be interpreted as evidence that an association is present only for boys or only for girls; that inference requires a direct interaction test.

### 4.2. Implications and Future Directions

These findings identify behaviors to distinguish in future evaluations, not instructions to increase or reduce a parent’s involvement. A useful test would compare similarly frequent homework assistance delivered with different levels of autonomy support, or similarly frequent conversations that differ in responsiveness. Evaluations should include both parents and assess adolescents’ experiences alongside academic outcomes and symptoms. Yap et al.’s (2016) meta-analysis of parent-focused preventive trials found very small average effects on depressive and internalizing symptoms across a broad age range. That evidence provides a reason to continue testing family-focused approaches. The outcomes of a brief communication intervention, however, cannot be inferred from the associations observed in this CEPS cohort alone.

Future longitudinal research could follow changes in both parents’ behaviors as adolescents encounter academic or emotional difficulties. Three or more waves with comparable measures could help distinguish stable between-family differences from within-person changes, a distinction emphasized by Hamaker et al. (2015). Diaries or observations could add information about whether students request help, who initiates conversations, and how responsibility is negotiated. Measuring parents’ work schedules and available time would also allow these possible constraints to be examined directly, instead of inferred from the identity of the responsible parent. Academic and psychological outcomes should remain part of the same study. Feeling supported may not coincide with higher examination scores, and an academically favorable association does not necessarily imply fewer symptoms. Including both domains permits direct assessment of these differences and avoids using a single outcome to represent adolescent adjustment as a whole.

### 4.3. Strengths and Limitations

A strength of this study is its joint analysis of four parent-specific behaviors, with cognitive assessments and school examination records considered alongside depressive symptoms. Corresponding baseline outcomes and class fixed effects account for measured prior functioning and shared classroom context. Direct contrasts test the comparative argument, and sensitivity analyses examine alternative samples, weighting, and measurement. The principal predictors and outcomes nevertheless come from the same follow-up wave. Unmeasured parental psychological well-being, emerging learning difficulties, and changing family relationships may influence both. Baseline adjustment cannot remove this confounding; the estimates describe between-adolescent associations and do not establish developmental changes caused by involvement.

Several measurement features limit the conceptual comparison. The single responsibility item does not capture the amount or quality of help and may be affected by differences in how respondents understand direct responsibility. Its test–retest reliability and agreement with independent measures of homework support were not assessed. Homework responsibility is parent-reported and communication is adolescent-reported, so behavioral domain and reporter are confounded. Differences between their coefficients cannot be assigned entirely to task-oriented versus relational processes. Standardization provides a common statistical scale, yet a standard-deviation difference in binary responsibility is not equivalent in effort or practical meaning to the same difference in communication frequency. Communication and symptoms also share an adolescent reporter, allowing reporting-related covariance. CEPS evidence of parent–adolescent discrepancies in perceived communication supports obtaining both perspectives while recognizing that they are not interchangeable (Zhang et al., 2021b). Alpha cannot resolve these reporter differences or establish construct validity. The supplementary factor analyses also showed poor single-factor fit for baseline communication and mixed fit for the symptom indices. These mean scores should therefore be interpreted as summaries of the specified communication topics or symptoms, rather than fully validated unidimensional constructs. Such measurement limitations may affect the estimated associations; their magnitude and direction cannot be determined from the factor analyses alone. In addition, communication and symptom item sets changed between waves, and the symptom index was not diagnostic. The class-standardized examination scores measure relative performance on school-administered tests. Differences in test content and possible recording inconsistencies remain relevant to their interpretation even after standardization.

Attrition and complete-case selection may also limit generalizability. Baseline differences indicate selection into the analytic samples. These comparisons do not identify the missing-data mechanism or the direction or magnitude of coefficient bias. Weighting and common-sample analyses cannot remove bias arising from unobserved reasons for nonparticipation or missing information; the unweighted primary estimates should not be read as nationally representative population estimates. Multiple testing warrants similar caution when selecting findings for emphasis, as several parent differences and configuration contrasts became less conclusive after adjustment. The second-order comparisons were exploratory additions rather than preregistered hypotheses. Finally, the cohort was observed in 2013–2015. Replication in more recent cohorts is needed before these patterns inform contemporary family or school programs, retaining the responsibility–communication distinction while improving measurement of the quality, content, and timing of both.

## 5. Conclusions

In this CEPS cohort, homework responsibility and parent–adolescent communication had small, outcome-specific associations conditional on baseline functioning. The maternal responsibility–communication contrasts remained significant across cognition, achievement, and depressive symptoms after multiple-testing adjustment. These concurrent associations do not identify benefits of increasing communication or reducing homework help. The study contributes a joint comparison of specific maternal and paternal behaviors across academic and psychological outcomes, showing why responsibility and communication should be distinguished without equating either with the quality of parenting.

## Figures and Tables

**Table 1:** Descriptive statistics for the common analytic sample.

Measure	N	Mean	SD	Range	α
Panel A. Continuous variables and multi-item scales					
Father–adolescent communication frequency	7245	1.94	0.55	1–3	0.82
Mother–adolescent communication frequency	7245	2.23	0.58	1–3	0.84
Cognitive ability	7245	0.09	0.95	−4.14–2.12	—
Academic achievement	7245	0.09	0.96	−4.80–2.71	—
Depressive symptoms	7245	2.15	0.89	1–5	0.92
Age	7245	13.51	0.68	12–18	—
Perceived family economic status	7245	2.96	0.59	1–5	—
Father’s educational attainment	7245	4.31	2.00	1–9	—
Mother’s educational attainment	7245	4.03	2.01	1–9	—
Panel B. Binary and multiple-response indicators					
Indicator	n (%)				
Father directly responsible for homework support	2180 (30.09%)				
Mother directly responsible for homework support	3005 (41.48%)				
Female	3645 (50.31%)				
Only child	3302 (45.58%)				
Non-agricultural/resident household registration	3475 (47.96%)				
Boarding student	2174 (30.01%)				

Notes: Statistics are calculated in the common complete-case sample used by all three fully adjusted outcome models. Panel A reports means and standard deviations to two decimal places; integer-valued scale endpoints are shown without trailing zeros. Panel B reports the number and percentage of adolescents meeting each indicator. Homework-support responsibility is a parent-reported multiple-response item; father and mother may both be identified. Cronbach’s alpha is calculated in the table sample for the four-item parent-specific communication measures and the six-item depressive-symptom measure. Higher depressive-symptom scores indicate poorer psychological adjustment. Means and standard deviations for perceived family economic status and parental educational attainment summarize the original category codes; these codes do not represent income amounts or years of schooling. Source: China Education Panel Survey (CEPS), 2013–2014 baseline and 2014–2015 follow-up.

**Table 2:** Correlations among parent-specific measures and adolescent outcomes.

Variable	1	2	3	4	5	6	7
1. Father directly responsible for homework support	1.000						
2. Mother directly responsible for homework support	0.253 ***	1.000					
3. Father–adolescent communication frequency	0.187 ***	0.100 ***	1.000				
4. Mother–adolescent communication frequency	0.082 ***	0.213 ***	0.572 ***	1.000			
5. Cognitive ability	0.041 ***	0.061 ***	0.106 ***	0.166 ***	1.000		
6. Academic achievement	−0.006	−0.032 **	0.082 ***	0.140 ***	0.413 ***	1.000	
7. Depressive symptoms	−0.079 ***	−0.049 ***	−0.154 ***	−0.142 ***	−0.077 ***	−0.064 ***	1.000

Notes: Pearson correlations in the common complete-case sample (N = 7245). All variables are standardized. Higher depressive-symptom values indicate poorer psychological adjustment. Statistical tests are two-sided. ** *p* < 0.01, *** *p* < 0.001. Stars refer to unadjusted, two-sided *p* values; results with *p* ≥ 0.05 receive no stars. Source: CEPS, 2013–2014 baseline and 2014–2015 follow-up.

**Table 3:** Parent-specific homework responsibility and communication: associations with cognitive ability.

	Dependent Variable: Cognitive Ability (Standardized)				
Predictor	(1)	(2)	(3)	(4)	(5)
Panel A. Regression estimates					
Father directly responsible for homework support	0.020	0.004	−0.018	−0.005	0.000
	(0.014)	(0.012)	(0.013)	(0.011)	(0.011)
Mother directly responsible for homework support	0.024	−0.020	−0.043 ***	−0.048 ***	−0.036 ***
	(0.015)	(0.012)	(0.011)	(0.010)	(0.010)
Father–adolescent communication frequency	0.006	0.010	0.002	−0.008	−0.008
	(0.017)	(0.017)	(0.016)	(0.012)	(0.011)
Mother–adolescent communication frequency	0.157 ***	0.121 ***	0.102 ***	0.065 ***	0.050 ***
	(0.020)	(0.018)	(0.017)	(0.013)	(0.011)
Child covariates	No	Yes	Yes	Yes	Yes
Family covariates	No	No	Yes	Yes	Yes
Class fixed effects	No	No	No	Yes	Yes
Lagged dependent variable	No	No	No	No	Yes
Observations	7604	7604	7604	7604	7604
R^2^	0.031	0.103	0.133	0.367	0.474
Panel B. Tests of coefficient equality (Model 5)					
Coefficient comparison				Unadjusted *p*	BH-adjusted *p* (*q*)
Father = mother: homework responsibility				0.033	0.056
Father = mother: communication				0.003	0.013
Responsibility = communication: father				0.654	0.714
Responsibility = communication: mother				<0.001	<0.001

Notes: Each column reports an OLS regression of standardized cognitive ability on the four parent-specific involvement measures entered jointly. All focal predictors are standardized; coefficients therefore represent the standard-deviation difference in the outcome associated with a one-standard-deviation difference in the predictor. Standard errors in parentheses are CR1 cluster-robust at the school level (112 schools); inference uses t(111) critical values. Child covariates are sex, age, only-child status, household registration, and boarding status. Family covariates are perceived economic status, maternal and paternal educational attainment, and parent co-residence. Column (5) adds the corresponding 2013–2014 lagged dependent variable; class fixed effects refer to 2014–2015 classes. The same complete-case sample is used in all columns (N = 7604; 295 classes). Cognitive ability is the CEPS three-parameter item-response-theory score from the 35-item follow-up test. Panel B reports two-sided coefficient-equality tests from Model 5: *p* values are unadjusted, and *q* values are Benjamini–Hochberg-adjusted across the 12 coefficient contrasts for the three outcomes. The adjusted threshold is *q* < 0.05. Appendix A Table A7 reports the differences and pointwise 95% confidence intervals, which are not multiplicity-adjusted. Tests are two-sided. *** *p* < 0.001. Stars refer to unadjusted, two-sided *p* values; results with *p* ≥ 0.05 receive no stars. Source: CEPS, 2013–2014 baseline and 2014–2015 follow-up.

**Table 4:** Parent-specific homework responsibility and communication: associations with academic achievement.

	Dependent Variable: Academic Achievement (Standardized)				
Predictor	(1)	(2)	(3)	(4)	(5)
Panel A. Regression estimates					
Father directly responsible for homework support	−0.004	−0.004	−0.017	−0.021	0.005
	(0.012)	(0.012)	(0.012)	(0.013)	(0.007)
Mother directly responsible for homework support	−0.062 ***	−0.059 ***	−0.065 ***	−0.065 ***	−0.020 *
	(0.012)	(0.012)	(0.012)	(0.012)	(0.008)
Father–adolescent communication frequency	−0.001	0.033 **	0.033 **	0.039 **	0.011
	(0.013)	(0.012)	(0.012)	(0.013)	(0.008)
Mother–adolescent communication frequency	0.151 ***	0.093 ***	0.087 ***	0.101 ***	0.041 ***
	(0.015)	(0.015)	(0.014)	(0.015)	(0.008)
Child covariates	No	Yes	Yes	Yes	Yes
Family covariates	No	No	Yes	Yes	Yes
Class fixed effects	No	No	No	Yes	Yes
Lagged dependent variable	No	No	No	No	Yes
Observations	7457	7457	7457	7457	7457
R^2^	0.024	0.089	0.095	0.119	0.695
Panel B. Tests of coefficient equality (Model 5)					
Coefficient comparison				Unadjusted *p*	BH-adjusted *p* (*q*)
Father = mother: homework responsibility				0.030	0.056
Father = mother: communication				0.037	0.056
Responsibility = communication: father				0.551	0.661
Responsibility = communication: mother				<0.001	<0.001

Notes: Each column reports an OLS regression of standardized academic achievement on the four parent-specific involvement measures entered jointly. All focal predictors are standardized; coefficients therefore represent the standard-deviation difference in the outcome associated with a one-standard-deviation difference in the predictor. Standard errors in parentheses are CR1 cluster-robust at the school level (112 schools); inference uses t(111) critical values. Child covariates are sex, age, only-child status, household registration, and boarding status. Family covariates are perceived economic status, maternal and paternal educational attainment, and parent co-residence. Column (5) adds the corresponding 2013–2014 lagged dependent variable; class fixed effects refer to 2014–2015 classes. The same complete-case sample is used in all columns (N = 7457; 290 classes). Academic achievement is the standardized mean of Chinese, mathematics, and English scores, with each subject first divided by its possible maximum and standardized within the follow-up class. Panel B reports two-sided coefficient-equality tests from Model 5: *p* values are unadjusted, and *q* values are Benjamini–Hochberg-adjusted across the 12 coefficient contrasts for the three outcomes. The adjusted threshold is *q* < 0.05. Appendix A Table A7 reports the differences and pointwise 95% confidence intervals, which are not multiplicity-adjusted. Tests are two-sided. * *p* < 0.05, ** *p* < 0.01, *** *p* < 0.001. Stars refer to unadjusted, two-sided *p* values; results with *p* ≥ 0.05 receive no stars. Source: CEPS, 2013–2014 baseline and 2014–2015 follow-up.

**Table 5:** Parent-specific homework responsibility and communication: associations with depressive symptoms.

	Dependent Variable: Depressive Symptoms (Standardized)				
Predictor	(1)	(2)	(3)	(4)	(5)
Panel A. Regression estimates					
Father directly responsible for homework support	−0.052 ***	−0.046 ***	−0.042 ***	−0.036 **	−0.036 **
	(0.012)	(0.012)	(0.012)	(0.013)	(0.011)
Mother directly responsible for homework support	−0.009	0.006	0.010	0.007	0.008
	(0.013)	(0.012)	(0.012)	(0.013)	(0.012)
Father–adolescent communication frequency	−0.097 ***	−0.092 ***	−0.087 ***	−0.085 ***	−0.054 ***
	(0.016)	(0.017)	(0.017)	(0.017)	(0.016)
Mother–adolescent communication frequency	−0.080 ***	−0.081 ***	−0.077 ***	−0.077 ***	−0.052 **
	(0.017)	(0.018)	(0.018)	(0.019)	(0.017)
Child covariates	No	Yes	Yes	Yes	Yes
Family covariates	No	No	Yes	Yes	Yes
Class fixed effects	No	No	No	Yes	Yes
Lagged dependent variable	No	No	No	No	Yes
Observations	7477	7477	7477	7477	7477
R^2^	0.031	0.039	0.046	0.111	0.256
Panel B. Tests of coefficient equality (Model 5)					
Coefficient comparison				Unadjusted *p*	BH-adjusted *p* (*q*)
Father = mother: homework responsibility				0.013	0.031
Father = mother: communication				0.941	0.941
Responsibility = communication: father				0.353	0.471
Responsibility = communication: mother				0.004	0.013

Notes: Each column reports an OLS regression of standardized depressive symptoms on the four parent-specific involvement measures entered jointly. All focal predictors are standardized; coefficients therefore represent the standard-deviation difference in the outcome associated with a one-standard-deviation difference in the predictor. Standard errors in parentheses are CR1 cluster-robust at the school level (112 schools); inference uses t(111) critical values. Child covariates are sex, age, only-child status, household registration, and boarding status. Family covariates are perceived economic status, maternal and paternal educational attainment, and parent co-residence. Column (5) adds the corresponding 2013–2014 lagged dependent variable; class fixed effects refer to 2014–2015 classes. The same complete-case sample is used in all columns (N = 7477; 295 classes). Depressive symptoms are the standardized mean of the first six CEPS C25 items assessing depressive affect during the previous seven days; higher values indicate more symptoms. Panel B reports two-sided coefficient-equality tests from Model 5: *p* values are unadjusted, and *q* values are Benjamini–Hochberg-adjusted across the 12 contrasts in Table 3, Table 4 and Table 5. The adjusted threshold is *q* < 0.05. Appendix A Table A7 reports the differences and pointwise 95% confidence intervals, which are not multiplicity-adjusted. Tests are two-sided. ** *p* < 0.01, *** *p* < 0.001. Stars refer to unadjusted, two-sided *p* values; results with *p* ≥ 0.05 receive no stars. Source: CEPS, 2013–2014 baseline and 2014–2015 follow-up.

**Table 6:** Sex differences in parent-specific associations.

	Standardized Association		
Predictor	Boys	Girls	*p* for Interaction
Panel A. Cognitive Ability			
Father directly responsible for homework support	−0.003	0.002	0.760
	(0.016)	(0.012)	
Mother directly responsible for homework support	−0.020	−0.051 ***	0.124
	(0.015)	(0.014)	
Father–adolescent communication frequency	−0.003	−0.011	0.705
	(0.015)	(0.015)	
Mother–adolescent communication frequency	0.050 **	0.049 ***	0.965
	(0.015)	(0.014)	
Observations	7604	7604	
Panel B. Academic Achievement			
Father directly responsible for homework support	0.008	0.002	0.673
	(0.011)	(0.009)	
Mother directly responsible for homework support	−0.023	−0.016	0.615
	(0.012)	(0.008)	
Father–adolescent communication frequency	0.025 *	−0.001	0.107
	(0.012)	(0.011)	
Mother–adolescent communication frequency	0.033 **	0.047 ***	0.353
	(0.012)	(0.011)	
Observations	7457	7457	
Panel C. Depressive Symptoms			
Father directly responsible for homework support	−0.038 *	−0.033 *	0.795
	(0.015)	(0.013)	
Mother directly responsible for homework support	0.013	0.005	0.699
	(0.016)	(0.016)	
Father–adolescent communication frequency	−0.045	−0.063 **	0.574
	(0.023)	(0.021)	
Mother–adolescent communication frequency	−0.045	−0.060 **	0.610
	(0.023)	(0.022)	
Observations	7477	7477	

Notes: Each panel reports sex-specific standardized associations from one fully adjusted OLS model containing all four parental-measure-by-sex interactions; these are not separate regressions for boys and girls. Boys are the reference group. Each estimate for girls is the sum of the corresponding main and interaction coefficients, with its standard error calculated from their joint covariance. The final column reports the two-sided *p* value for the interaction, directly testing the difference between boys and girls. All models include the four parental measures, child and family covariates, the corresponding baseline outcome, and follow-up class fixed effects. Observations denote the pooled sample, not sex-specific counts. CR1 school-clustered standard errors are in parentheses (112 schools). * *p* < 0.05, ** *p* < 0.01, *** *p* < 0.001. Stars refer to unadjusted, two-sided *p* values; results with *p* ≥ 0.05 receive no stars. Stars indicate within-sex associations, not tests of differences between boys and girls. Source: CEPS, 2013–2014 baseline and 2014–2015 follow-up.

## Data Availability

The data used in this study are available through the social science data platform of Renmin University of China (https://cssd.ruc.edu.cn/home (accessed on 9 October 2026)) by searching for “China Education Panel Survey.” The project numbers for the 2013–2014 and 2014–2015 waves in the Chinese National Survey Data Archive (CNSDA) are 72810330 and 61662993, respectively. Access is subject to the platform’s registration and data-use requirements. Further information is available on the CEPS website (http://ceps.ruc.edu.cn/ (accessed on 9 October 2026)).

## Appendix A

**Table A1:** Sample selection and missingness.

Panel A. Sequential Selection of the Analytic Samples				
Eligibility Requirement	Retained n [Additional Exclusions]			
	Cognitive Ability	Academic Achievement	Depressive Symptoms	Common Sample
Original seventh-grade baseline cohort	10,279	10,279	10,279	10,279
Successfully followed in 2014–2015	9449 [830]	9449 [830]	9449 [830]	9449 [830]
Both homework-responsibility indicators observed	9149 [300]	9149 [300]	9149 [300]	9149 [300]
Both communication scores observed	8687 [462]	8687 [462]	8687 [462]	8687 [462]
Follow-up outcome(s) observed	8626 [61]	8581 [106]	8679 [8]	8517 [170]
Corresponding baseline outcome(s) observed	8626 [0]	8449 [132]	8467 [212]	8186 [331]
All child covariates observed	7900 [726]	7749 [700]	7762 [705]	7525 [661]
All family covariates observed	7604 [296]	7457 [292]	7477 [285]	7245 [280]
Follow-up class and school identifiers observed	7604 [0]	7457 [0]	7477 [0]	7245 [0]
Longitudinal weight observed: final analytic sample	7604 [0]	7457 [0]	7477 [0]	7245 [0]
**Panel B. Missing Required Variables Among Successfully Followed Adolescents (N = 9449)**				
**Required Variable**			**Available n**	**Missing n (%)**
Parental measures at follow-up				
Father directly responsible for homework support			9149	300 (3.17)
Mother directly responsible for homework support			9149	300 (3.17)
Father–adolescent communication frequency			9279	170 (1.80)
Mother–adolescent communication frequency			9086	363 (3.84)
Outcomes and corresponding baseline measures				
Cognitive ability: follow-up			9366	83 (0.88)
Cognitive ability: baseline			9449	0 (0.00)
Academic achievement: follow-up			9318	131 (1.39)
Academic achievement: baseline			9264	185 (1.96)
Depressive symptoms: follow-up			9420	29 (0.31)
Depressive symptoms: baseline			9204	245 (2.59)
Child covariates				
Female			9449	0 (0.00)
Age			9234	215 (2.28)
Only child			9306	143 (1.51)
Non-agricultural/resident household registration			9100	349 (3.69)
Boarding student			9242	207 (2.19)
Family covariates				
Perceived family economic status			9394	55 (0.58)
Father’s educational attainment			9064	385 (4.07)
Mother’s educational attainment			9032	417 (4.41)
Father co-resident			9366	83 (0.88)
Mother co-resident			9366	83 (0.88)
Identifiers and sampling weight				
Follow-up class identifier			9449	0 (0.00)
School identifier			9449	0 (0.00)
Longitudinal sampling weight			9449	0 (0.00)

Notes A: Cells report the number retained after each requirement; brackets show additional exclusions at that step. The 471 newly sampled Wave 2 students were outside the original cohort and are not counted as attrition. Exclusions are mutually exclusive within the stated sequence; the three outcome-specific samples overlap. The common sample requires complete data for all three outcomes and their baseline counterparts. Child covariates are sex, age, only-child status, household registration, and boarding status; family covariates are perceived economic status, parental education, and parent co-residence. The observed-weight requirement applies to both unweighted primary and weighted sensitivity models so that their samples are comparable. Zero additional exclusions at a later step do not imply that the variable has no missing observations. Source: CEPS, 2013–2014 baseline and 2014–2015 follow-up. Notes B: The denominator is 9449 for every row. Missingness refers to unavailable analysis variables after applying the scoring and validity rules used in the analyses, including insufficient valid scale items; it is not restricted to blank raw responses. Row counts overlap and must not be added. Each model requires only its own outcome and corresponding baseline measure. Source: CEPS, 2013–2014 baseline and 2014–2015 follow-up.

**Table A2:** Baseline characteristics by analytic-sample inclusion and follow-up status.

Panel A. Cognitive Ability						
Baseline Characteristic	Included (n = 7604)		Excluded (n = 1845)		SMD	*p*
	Available n	Mean (SD)/ n (%)	Available n	Mean (SD)/ n (%)		
Female	7604	3778 (49.68)	1845	734 (39.78)	0.200	<0.001
Age (years)	7604	12.52 (0.69)	1630	12.65 (0.76)	−0.180	<0.001
Only child	7604	3458 (45.48)	1845	757 (41.03)	0.090	0.012
Non-agricultural household registration	7604	3650 (48.00)	1845	844 (45.75)	0.045	0.242
Boarding student	7604	2300 (30.25)	1845	560 (30.35)	−0.002	0.954
Father co-resident	7604	6198 (81.51)	1845	1389 (75.28)	0.152	<0.001
Mother co-resident	7604	6626 (87.14)	1845	1498 (81.19)	0.163	<0.001
Perceived family economic status						
Difficult	7592	1534 (20.21)	1833	451 (24.60)	−0.106	0.001
Average	7592	5598 (73.74)	1833	1288 (70.27)	0.077	0.008
Affluent	7592	460 (6.06)	1833	94 (5.13)	0.041	0.121
Father’s educational attainment						
Primary school or below	7594	1004 (13.22)	1835	299 (16.29)	−0.087	0.003
Junior secondary	7594	3233 (42.57)	1835	798 (43.49)	−0.018	0.581
Upper secondary	7594	2063 (27.17)	1835	475 (25.89)	0.029	0.320
College or above	7594	1294 (17.04)	1835	263 (14.33)	0.074	0.047
Mother’s educational attainment						
Primary school or below	7594	1619 (21.32)	1835	452 (24.63)	−0.079	0.010
Junior secondary	7594	3114 (41.01)	1835	767 (41.80)	−0.016	0.567
Upper secondary	7594	1763 (23.22)	1835	412 (22.45)	0.018	0.450
College or above	7594	1098 (14.46)	1835	204 (11.12)	0.100	0.005
Father–adolescent communication frequency	7220	1.95 (0.58)	1640	1.90 (0.59)	0.092	0.003
Mother–adolescent communication frequency	7460	2.17 (0.56)	1768	2.07 (0.59)	0.175	<0.001
Cognitive ability (standardized)	7604	0.09 (0.98)	1845	−0.17 (1.03)	0.255	<0.001
Academic achievement (standardized)	7471	0.10 (0.94)	1793	−0.24 (1.10)	0.323	<0.001
Depressive symptoms	7433	1.99 (0.77)	1771	2.06 (0.86)	−0.093	<0.001
**Panel B. Academic Achievement**						
**Baseline Characteristic**	**Included (n = 7457)**		**Excluded (n = 1992)**		**SMD**	***p***
	**Available** **n**	**Mean (SD)/** **n (%)**	**Available** **n**	**Mean (SD)/** **n (%)**		
Female	7457	3722 (49.91)	1992	790 (39.66)	0.207	<0.001
Age (years)	7457	12.52 (0.69)	1777	12.67 (0.76)	−0.211	<0.001
Only child	7457	3420 (45.86)	1992	795 (39.91)	0.121	<0.001
Non-agricultural household registration	7457	3596 (48.22)	1992	898 (45.08)	0.063	0.113
Boarding student	7457	2248 (30.15)	1992	612 (30.72)	−0.013	0.764
Father co-resident	7457	6081 (81.55)	1992	1506 (75.60)	0.145	<0.001
Mother co-resident	7457	6501 (87.18)	1992	1623 (81.48)	0.157	<0.001
Perceived family economic status						
Difficult	7447	1503 (20.18)	1978	482 (24.37)	−0.101	0.002
Average	7447	5488 (73.69)	1978	1398 (70.68)	0.067	0.020
Affluent	7447	456 (6.12)	1978	98 (4.95)	0.051	0.052
Father’s educational attainment						
Primary school or below	7448	979 (13.14)	1981	324 (16.36)	−0.091	0.002
Junior secondary	7448	3168 (42.53)	1981	863 (43.56)	−0.021	0.533
Upper secondary	7448	2022 (27.15)	1981	516 (26.05)	0.025	0.381
College or above	7448	1279 (17.17)	1981	278 (14.03)	0.087	0.021
Mother’s educational attainment						
Primary school or below	7448	1573 (21.12)	1981	498 (25.14)	−0.095	0.005
Junior secondary	7448	3051 (40.96)	1981	830 (41.90)	−0.019	0.515
Upper secondary	7448	1733 (23.27)	1981	442 (22.31)	0.023	0.334
College or above	7448	1091 (14.65)	1981	211 (10.65)	0.120	<0.001
Father–adolescent communication frequency	7094	1.95 (0.58)	1766	1.90 (0.59)	0.093	<0.001
Mother–adolescent communication frequency	7323	2.18 (0.56)	1905	2.07 (0.58)	0.186	<0.001
Cognitive ability (standardized)	7457	0.10 (0.98)	1992	−0.19 (1.03)	0.283	<0.001
Academic achievement (standardized)	7457	0.10 (0.94)	1807	−0.24 (1.11)	0.329	<0.001
Depressive symptoms	7291	1.98 (0.77)	1913	2.07 (0.86)	−0.105	<0.001
**Panel C. Depressive Symptoms**						
**Baseline Characteristic**	**Included (n = 7477)**		**Excluded (n = 1972)**		**SMD**	***p***
	**Available** **n**	**Mean (SD)/** **n (%)**	**Available** **n**	**Mean (SD)/** **n (%)**		
Female	7477	3739 (50.01)	1972	773 (39.20)	0.219	<0.001
Age (years)	7477	12.52 (0.69)	1757	12.65 (0.77)	−0.181	<0.001
Only child	7477	3404 (45.53)	1972	811 (41.13)	0.089	0.007
Non-agricultural household registration	7477	3586 (47.96)	1972	908 (46.04)	0.038	0.316
Boarding student	7477	2263 (30.27)	1972	597 (30.27)	−0.000	0.997
Father co-resident	7477	6099 (81.57)	1972	1488 (75.46)	0.149	<0.001
Mother co-resident	7477	6526 (87.28)	1972	1598 (81.03)	0.172	<0.001
Perceived family economic status						
Difficult	7466	1488 (19.93)	1959	497 (25.37)	−0.130	<0.001
Average	7466	5524 (73.99)	1959	1362 (69.53)	0.099	<0.001
Affluent	7466	454 (6.08)	1959	100 (5.10)	0.042	0.100
Father’s educational attainment						
Primary school or below	7469	981 (13.13)	1960	322 (16.43)	−0.093	<0.001
Junior secondary	7469	3172 (42.47)	1960	859 (43.83)	−0.027	0.384
Upper secondary	7469	2039 (27.30)	1960	499 (25.46)	0.042	0.147
College or above	7469	1277 (17.10)	1960	280 (14.29)	0.077	0.032
Mother’s educational attainment						
Primary school or below	7469	1579 (21.14)	1960	492 (25.10)	−0.094	0.001
Junior secondary	7469	3056 (40.92)	1960	825 (42.09)	−0.024	0.388
Upper secondary	7469	1747 (23.39)	1960	428 (21.84)	0.037	0.111
College or above	7469	1087 (14.55)	1960	215 (10.97)	0.108	0.002
Father–adolescent communication frequency	7121	1.95 (0.58)	1739	1.90 (0.58)	0.087	0.004
Mother–adolescent communication frequency	7355	2.18 (0.56)	1873	2.07 (0.58)	0.192	<0.001
Cognitive ability (standardized)	7477	0.09 (0.98)	1972	−0.18 (1.03)	0.274	<0.001
Academic achievement (standardized)	7348	0.11 (0.93)	1916	−0.27 (1.12)	0.371	<0.001
Depressive symptoms	7477	1.99 (0.77)	1727	2.06 (0.87)	−0.093	0.001
**Panel D. Follow-Up Status in the Original Baseline Cohort**						
**Baseline Characteristic**	**Followed (n = 9449)**		**Not Followed (n = 830)**		**SMD**	***p***
	**Available** **n**	**Mean (SD)/** **n (%)**	**Available** **n**	**Mean (SD)/** **n (%)**		
Female	9449	4512 (47.75)	830	340 (40.96)	0.137	<0.001
Age (years)	9234	12.55 (0.71)	811	12.94 (0.92)	−0.485	<0.001
Only child	9449	4215 (44.61)	830	202 (24.34)	0.437	<0.001
Non-agricultural household registration	9449	4494 (47.56)	830	253 (30.48)	0.356	<0.001
Boarding student	9449	2860 (30.27)	830	327 (39.40)	−0.193	0.055
Father co-resident	9449	7587 (80.29)	830	591 (71.20)	0.213	<0.001
Mother co-resident	9449	8124 (85.98)	830	627 (75.54)	0.267	<0.001
Perceived family economic status						
Difficult	9425	1985 (21.06)	821	218 (26.55)	−0.129	0.009
Average	9425	6886 (73.06)	821	556 (67.72)	0.117	0.013
Affluent	9425	554 (5.88)	821	47 (5.72)	0.007	0.875
Father’s educational attainment						
Primary school or below	9429	1303 (13.82)	823	176 (21.39)	−0.200	<0.001
Junior secondary	9429	4031 (42.75)	823	394 (47.87)	−0.103	0.008
Upper secondary	9429	2538 (26.92)	823	183 (22.24)	0.109	0.014
College or above	9429	1557 (16.51)	823	70 (8.51)	0.244	<0.001
Mother’s educational attainment						
Primary school or below	9429	2071 (21.96)	823	277 (33.66)	−0.263	<0.001
Junior secondary	9429	3881 (41.16)	823	346 (42.04)	−0.018	0.670
Upper secondary	9429	2175 (23.07)	823	142 (17.25)	0.145	0.002
College or above	9429	1302 (13.81)	823	58 (7.05)	0.223	<0.001
Father–adolescent communication frequency	8860	1.94 (0.58)	727	1.88 (0.60)	0.102	0.033
Mother–adolescent communication frequency	9228	2.16 (0.57)	787	2.02 (0.59)	0.228	<0.001
Cognitive ability (standardized)	9449	0.04 (1.00)	830	−0.40 (0.94)	0.448	<0.001
Academic achievement (standardized)	9264	0.03 (0.98)	773	−0.37 (1.14)	0.379	<0.001
Depressive symptoms	9204	2.00 (0.79)	792	2.17 (0.86)	−0.211	<0.001

Notes: Panels A–C compare inclusion in the corresponding fully adjusted model among the 9449 successfully followed adolescents; the 830 non-followed students are not included. Panel D compares successfully followed and non-followed adolescents within the original 10,279-person seventh-grade cohort; it does not compare complete-case eligibility. All characteristics were measured at baseline. Values are unweighted means (SDs) or n (%), using the available n shown for each group. SMD is the signed standardized mean difference (left group minus right), with the square root of the average group variances as denominator; binary variances are calculated from within-group proportions. Two-sided *p* values test group differences using OLS with school-clustered CR1 standard errors (112 schools; t(111) inference). They are descriptive and unadjusted for multiple testing. Category rows are not omnibus tests. Age is 2013 minus birth year. Communication scores range from 1 to 3 and depressive symptoms from 1 to 5. Cognitive ability and achievement were standardized using valid observations in the linked baseline cohort. Family economic status uses the released three-category baseline measure. Upper secondary includes vocational and technical education; college includes junior college and higher degrees. Education is grouped here for descriptive comparisons only. Source: CEPS, 2013–2014 baseline and 2014–2015 follow-up.

**Table A3:** Sensitivity and temporal-ordering estimates.

Outcome	Father Directly Responsible for Homework Support	Mother Directly Responsible for Homework Support	Father–Adolescent Communication Frequency	Mother–Adolescent Communication Frequency	N
Panel A. Longitudinal survey weighted					
Cognitive ability	−0.015 (0.012)	−0.037 ** (0.012)	−0.034 * (0.017)	0.078 *** (0.012)	7604
Academic achievement	−0.001 (0.009)	−0.026 ** (0.009)	0.019 (0.011)	0.041 ** (0.012)	7457
Depressive symptoms	−0.057 *** (0.014)	0.027 (0.017)	−0.049 * (0.019)	−0.049 * (0.023)	7477
Panel B. Common three-outcome sample					
Cognitive ability	0.000 (0.011)	−0.038 *** (0.010)	−0.005 (0.011)	0.047 *** (0.011)	7245
Academic achievement	0.006 (0.007)	−0.020 * (0.008)	0.010 (0.008)	0.042 *** (0.009)	7245
Depressive symptoms	−0.036 *** (0.011)	0.008 (0.011)	−0.051 ** (0.016)	−0.053 ** (0.017)	7245
Panel C. Both biological parents co-resident					
Cognitive ability	0.002 (0.011)	−0.033 ** (0.011)	−0.007 (0.013)	0.050 *** (0.011)	6192
Academic achievement	0.012 (0.007)	−0.021 ** (0.008)	0.010 (0.009)	0.048 *** (0.009)	6083
Depressive symptoms	−0.036 ** (0.012)	0.014 (0.013)	−0.042 * (0.019)	−0.069 ** (0.020)	6091
Panel D. Baseline communication (prospective)					
Cognitive ability	—	—	−0.021 * (0.009)	0.040 *** (0.011)	7499
Academic achievement	—	—	−0.003 (0.009)	0.031 *** (0.008)	7364
Depressive symptoms	—	—	−0.006 (0.015)	−0.029 (0.015)	7395
Panel E. Alternative achievement: mean score-to-maximum ratio					
Academic achievement	0.006 (0.005)	−0.012 (0.006)	0.003 (0.006)	0.033 *** (0.006)	7458

Notes: Each outcome row reports one fully adjusted model. Cells show standardized coefficients with school-clustered CR1 standard errors below in parentheses (112 schools; t(111) inference). All models include child and family covariates, follow-up class fixed effects, and the corresponding baseline outcome. Child covariates are sex, age, only-child status, household registration, and boarding status; family covariates are perceived economic status, parental education, and parent co-residence. Panel A uses longitudinal-weighted least squares; Panels B–E use unweighted OLS. Panels A–C and E jointly include all four parental measures. Panel D instead includes the two baseline communication scores and omits follow-up homework responsibility; dashes denote omitted predictors. Panel E uses the standardized mean score-to-maximum ratio as the achievement outcome. Tests are two-sided and *p* values are unadjusted. * *p* < 0.05, ** *p* < 0.01, *** *p* < 0.001. Stars refer to unadjusted, two-sided *p* values; results with *p* ≥ 0.05 receive no stars. Source: CEPS, 2013–2014 baseline and 2014–2015 follow-up.

**Table A4:** Sensitivity of parental associations to the coding of socioeconomic covariates.

Predictor	(1) Numeric Ordered Coding	(2) Categorical Coding
Panel A. Cognitive ability		
Father directly responsible for homework support	0.000	−0.001
	(0.011)	(0.011)
Mother directly responsible for homework support	−0.036 ***	−0.038 ***
	(0.010)	(0.010)
Father–adolescent communication frequency	−0.008	−0.006
	(0.011)	(0.011)
Mother–adolescent communication frequency	0.050 ***	0.049 ***
	(0.011)	(0.011)
Observations	7604	7604
R^2^	0.474	0.478
Panel B. Academic achievement		
Father directly responsible for homework support	0.005	0.006
	(0.007)	(0.007)
Mother directly responsible for homework support	−0.020 *	−0.020 *
	(0.008)	(0.008)
Father–adolescent communication frequency	0.011	0.010
	(0.008)	(0.008)
Mother–adolescent communication frequency	0.041 ***	0.041 ***
	(0.008)	(0.008)
Observations	7457	7457
R^2^	0.695	0.696
Panel C. Depressive symptoms		
Father directly responsible for homework support	−0.036 **	−0.035 **
	(0.011)	(0.011)
Mother directly responsible for homework support	0.008	0.007
	(0.012)	(0.012)
Father–adolescent communication frequency	−0.054 ***	−0.054 ***
	(0.016)	(0.016)
Mother–adolescent communication frequency	−0.052 **	−0.053 **
	(0.017)	(0.017)
Observations	7477	7477
R^2^	0.256	0.257

Notes: Within each panel, columns (1) and (2) report separate fully adjusted, unweighted OLS regressions. The four parental measures are entered jointly; the predictors shown and the outcomes are standardized. Standard errors in parentheses are CR1 cluster-robust at the school level (112 schools); inference uses a *t* distribution with 111 degrees of freedom. Column (1) enters family economic conditions (1–5) and each parent’s educational attainment (1–9) as numeric ordered covariates. Column (2) replaces these three terms with category indicators, using “very difficult” economic conditions and “no schooling” as the respective reference categories; no categories are combined. Both specifications adjust for sex, age, only-child status, household registration, boarding status, co-residence with each parent, the corresponding 2013–2014 outcome, and 2014–2015 class fixed effects. Within each panel, the complete-case sample and all standardization procedures are identical across columns. Higher depressive-symptom scores indicate poorer psychological adjustment. Asterisks refer to two-sided, unadjusted *p* values: * *p* < 0.05, ** *p* < 0.01, *** *p* < 0.001. Source: CEPS, 2013–2014 baseline and 2014–2015 follow-up.

**Table A5:** Subject-specific associations with academic achievement.

	Dependent Variable: Subject-Specific Academic Achievement (Standardized)		
Predictor	Chinese	Mathematics	English
Father directly responsible for homework support	−0.002	−0.004	0.007
	(0.008)	(0.010)	(0.008)
Mother directly responsible for homework support	−0.018	−0.025 **	−0.028 **
	(0.009)	(0.009)	(0.008)
Father–adolescent communication frequency	0.011	0.014	0.016
	(0.009)	(0.012)	(0.009)
Mother–adolescent communication frequency	0.042 ***	0.060 ***	0.033 **
	(0.011)	(0.011)	(0.010)
Child and family covariates	Yes	Yes	Yes
Class fixed effects	Yes	Yes	Yes
Subject-specific lagged outcome	Yes	Yes	Yes
Observations	7471	7468	7467
R^2^	0.484	0.493	0.601

Notes: Each column reports a separate fully adjusted OLS regression of a class-standardized subject score on the four parent-specific involvement measures entered jointly. Raw follow-up scores are divided by the relevant possible maximum and standardized within the 2014–2015 class. Each model includes child and family covariates, 2014–2015 class fixed effects, and the corresponding 2013–2014 subject score. All focal predictors are standardized. Standard errors in parentheses are CR1 cluster-robust at the school level (112 schools); inference uses t(111) critical values. Statistical tests are two-sided. ** *p* < 0.01, *** *p* < 0.001. Stars refer to unadjusted, two-sided *p* values; results with *p* ≥ 0.05 receive no stars. Source: CEPS, 2013–2014 baseline and 2014–2015 follow-up.

**Table A6:** Homework-responsibility configurations and adolescent adjustment.

Configuration/Contrast	Cognitive Ability	Academic Achievement	Depressive Symptoms
Panel A. Sample distribution, n (%)			
Neither parent identified	3564 (46.9)	3482 (46.7)	3501 (46.8)
Father only identified	908 (11.9)	887 (11.9)	893 (11.9)
Mother only identified	1757 (23.1)	1728 (23.2)	1727 (23.1)
Both parents identified	1375 (18.1)	1360 (18.2)	1356 (18.1)
Observations	7604	7457	7477
School clusters	112	112	112
Panel B. Adjusted associations relative to neither parent identified			
Father only identified	−0.002 [−0.058, 0.054] *p*/*q* = 0.938/0.951	−0.024 [−0.067, 0.019] *p*/*q* = 0.276/0.414	−0.088 * [−0.156, −0.019] *p*/*q* = 0.012/0.052
Mother only identified	−0.075 ** [−0.121, −0.028] *p*/*q* = 0.002/0.021	−0.062 ** [−0.100, −0.024] *p*/*q* = 0.002/0.021	0.011 [−0.043, 0.065] *p*/*q* = 0.681/0.883
Both parents identified	−0.073 ** [−0.126, −0.020] *p*/*q* = 0.007/0.0505	−0.022 [−0.062, 0.018] *p*/*q* = 0.273/0.414	−0.060 * [−0.118, −0.002] *p*/*q* = 0.043/0.089
Panel C. Adjusted contrasts between configurations			
Both parents identified − father only identified	−0.071 * [−0.128, −0.013] *p*/*q* = 0.016/0.057	0.002 [−0.043, 0.046] *p*/*q* = 0.935/0.951	0.028 [−0.049, 0.104] *p*/*q* = 0.477/0.667
Both parents identified − mother only identified	0.002 [−0.055, 0.059] *p*/*q* = 0.951/0.951	0.040 * [0.001, 0.079] *p*/*q* = 0.047/0.089	−0.071 * [−0.131, −0.011] *p*/*q* = 0.020/0.061
Father only identified − mother only identified	0.072 * [0.002, 0.143] *p*/*q* = 0.043/0.089	0.038 [−0.009, 0.085] *p*/*q* = 0.114/0.200	−0.099 * [−0.175, −0.022] *p*/*q* = 0.012/0.052
Departure from additivity	0.004 [−0.060, 0.068] *p*/*q* = 0.903/0.951	0.063 * [0.008, 0.119] *p*/*q* = 0.025/0.065	0.016 [−0.072, 0.105] *p*/*q* = 0.715/0.883

Notes: CEPS 2013–2014 and 2014–2015 linked cohort; outcome-specific complete-case samples. “Identified” means selected in the multiple-response parent-questionnaire item A15 as directly responsible for homework/study support. “Only” refers to the two parents: an unselected parent may still help, and other family members may be responsible in every configuration. Panel A reports unweighted counts and within-outcome percentages. Panels B–C report estimates, pointwise 95% confidence intervals in brackets, and two-sided *p*/*q* values from unweighted OLS models adjusting for both communication measures, child and family covariates, the corresponding baseline outcome, and follow-up class fixed effects. Estimates are in outcome-standard-deviation units for raw configuration contrasts; configuration indicators are not standardized. Inference uses CR1 school-clustered standard errors and t(111). Departure from additivity equals the both-parents coefficient minus the father-only and mother-only coefficients, each relative to neither parent identified. *p* values are unadjusted; *q* values are Benjamini–Hochberg-adjusted *p* values across all 21 tests in Panels B–C. The borderline *q* value is shown to four decimals. Confidence intervals are not multiplicity-adjusted; stars refer to unadjusted *p* values. * *p* < 0.05, ** *p* < 0.01. Stars refer to unadjusted, two-sided *p* values; results with *p* ≥ 0.05 receive no stars. The primary inferential threshold remains *p* < 0.05.

**Table A7:** Direct coefficient comparisons in the fully adjusted models.

Coefficient Comparison	Δ*β*	95% CI	*p*	*q* (BH)
Panel A. Cognitive Ability (N = 7604)				
Father − mother: homework responsibility	0.036	[0.003, 0.069]	0.033	0.056
Father − mother: communication frequency	−0.057	[−0.095, −0.020]	0.003	0.013
Homework responsibility − communication: father	0.007	[−0.026, 0.041]	0.654	0.714
Homework responsibility − communication: mother	−0.085	[−0.115, −0.056]	<0.001	<0.001
Panel B. Academic Achievement (N = 7457)				
Father − mother: homework responsibility	0.024	[0.002, 0.046]	0.030	0.056
Father − mother: communication frequency	−0.030	[−0.058, −0.002]	0.037	0.056
Homework responsibility − communication: father	−0.007	[−0.030, 0.016]	0.551	0.661
Homework responsibility − communication: mother	−0.061	[−0.086, −0.036]	<0.001	<0.001
Panel C. Depressive Symptoms (N = 7477)				
Father − mother: homework responsibility	−0.044	[−0.079, −0.010]	0.013	0.031
Father − mother: communication frequency	−0.002	[−0.059, 0.055]	0.941	0.941
Homework responsibility − communication: father	0.018	[−0.020, 0.056]	0.353	0.471
Homework responsibility − communication: mother	0.060	[0.019, 0.101]	0.004	0.013
Panel D. Exploratory second-order comparisons				
Cognitive Ability	−0.093	[−0.146, −0.040]	<0.001	0.002
Academic Achievement	−0.054	[−0.093, −0.015]	0.007	0.011
Depressive Symptoms	0.042	[−0.026, 0.110]	0.221	0.221

Notes: FH and MH denote father and mother directly responsible for homework support; FC and MC denote father–adolescent and mother–adolescent communication frequency. In Panels A–C, Δ*β* is the first coefficient minus the second. Panel D reports (MH − MC) − (FH − FC) for the same outcome-specific samples. Estimates are linear combinations from Model 5 of Table 3, Table 4 and Table 5, using unweighted OLS with all four focal predictors entered jointly, child and family covariates, the corresponding baseline outcome, and 2014–2015 class fixed effects. Child covariates are sex, age, only-child status, household registration, and boarding status; family covariates are perceived economic status, parental educational attainment, and parent co-residence. Predictors and outcomes use the original linked-cohort standardization; differences concern standardized associations, not equivalent amounts or qualities of involvement. Confidence intervals are pointwise 95% t intervals from the full school-clustered CR1 covariance matrix (112 schools; 111 degrees of freedom). *p* values are two-sided and unadjusted. *q* values apply Benjamini–Hochberg adjustment across the 12 comparisons in Panels A–C and, separately, the three exploratory comparisons in Panel D. The confidence intervals are not multiplicity-adjusted. Source: CEPS, 2013–2014 baseline grade-7 cohort and 2014–2015 follow-up.

## References

[B1-behavsci-16-01898] Barger M. M., Kim E. M., Kuncel N. R., Pomerantz E. M. (2019). The relation between parents’ involvement in children’s schooling and children’s adjustment: A meta-analysis. Psychological Bulletin.

[B2-behavsci-16-01898] Benjamini Y., Hochberg Y. (1995). Controlling the false discovery rate: A practical and powerful approach to multiple testing. Journal of the Royal Statistical Society: Series B (Methodological).

[B3-behavsci-16-01898] Boonk L., Gijselaers H. J. M., Ritzen H., Brand-Gruwel S. (2018). A review of the relationship between parental involvement indicators and academic achievement. Educational Research Review.

[B4-behavsci-16-01898] Cameron A. C., Miller D. L. (2015). A practitioner’s guide to cluster-robust inference. Journal of Human Resources.

[B5-behavsci-16-01898] Cheung C. S.-S., Pomerantz E. M. (2011). Parents’ involvement in children’s learning in the United States and China: Implications for children’s academic and emotional adjustment. Child Development.

[B6-behavsci-16-01898] China Education Panel Survey Project Team (2015). China Education Panel Survey (CEPS) baseline data user manual.

[B7-behavsci-16-01898] China Education Panel Survey Project Team (2017). China Education Panel Survey (CEPS) 2014–2015 follow-up data user manual.

[B8-behavsci-16-01898] Cronbach L. J. (1951). Coefficient alpha and the internal structure of tests. Psychometrika.

[B9-behavsci-16-01898] Cui T., Liu Q. (2025). Parental involvement and academic achievement in Year 4 and Year 8 students: A meta-analysis based on large-scale assessments in China. British Educational Research Journal.

[B10-behavsci-16-01898] Duan W., Guan Y., Bu H. (2018). The effect of parental involvement and socioeconomic status on junior school students’ academic achievement and school behavior in China. Frontiers in Psychology.

[B11-behavsci-16-01898] Dumont H., Trautwein U., Nagy G., Nagengast B. (2014). Quality of parental homework involvement: Predictors and reciprocal relations with academic functioning in the reading domain. Journal of Educational Psychology.

[B12-behavsci-16-01898] Feinberg M. E. (2003). The internal structure and ecological context of coparenting: A framework for research and intervention. Parenting: Science and Practice.

[B13-behavsci-16-01898] Gelman A., Stern H. (2006). The difference between “significant” and “not significant” is not itself statistically significant. The American Statistician.

[B14-behavsci-16-01898] Grolnick W. S., Slowiaczek M. L. (1994). Parents’ involvement in children’s schooling: A multidimensional conceptualization and motivational model. Child Development.

[B15-behavsci-16-01898] Hamaker E. L., Kuiper R. M., Grasman R. P. P. P. (2015). A critique of the cross-lagged panel model. Psychological Methods.

[B16-behavsci-16-01898] Hill N. E., Castellino D. R., Lansford J. E., Nowlin P., Dodge K. A., Bates J. E., Pettit G. S. (2004). Parent academic involvement as related to school behavior, achievement, and aspirations: Demographic variations across adolescence. Child Development.

[B17-behavsci-16-01898] Hill N. E., Tyson D. F. (2009). Parental involvement in middle school: A meta-analytic assessment of the strategies that promote achievement. Developmental Psychology.

[B18-behavsci-16-01898] Hou J., Chen Z., Guo F. (2020). The transactional relationship between parental and adolescent depressive symptoms: The mediating effect of nurturant-involved parenting. International Journal of Environmental Research and Public Health.

[B19-behavsci-16-01898] Hsu H.-Y., Zhang D., Kwok O.-M., Li Y., Ju S. (2011). Distinguishing the influences of father’s and mother’s involvement on adolescent academic achievement: Analyses of Taiwan Education Panel Survey data. The Journal of Early Adolescence.

[B20-behavsci-16-01898] Hu J., Zhou T. (2025). Parent- and adolescent-driven effects in emotion-related communication and longitudinal relationships with depressive symptoms in adolescents. Journal of Youth and Adolescence.

[B21-behavsci-16-01898] Jeynes W. H. (2015). A meta-analysis: The relationship between father involvement and student academic achievement. Urban Education.

[B22-behavsci-16-01898] Jiang Q., Shi L., Zheng D., Mao W. (2023). Parental homework involvement and students’ mathematics achievement: A meta-analysis. Frontiers in Psychology.

[B23-behavsci-16-01898] Kang L., Li C., Chen D., Bao X. (2024). Parental involvement, academic self-efficacy, and depression on academic performance among Chinese students during COVID-19 pandemic. Psychology Research and Behavior Management.

[B24-behavsci-16-01898] Kerr M., Stattin H. (2000). What parents know, how they know it, and several forms of adolescent adjustment: Further support for a reinterpretation of monitoring. Developmental Psychology.

[B25-behavsci-16-01898] Kim S. (2022). Fifty years of parental involvement and achievement research: A second-order meta-analysis. Educational Research Review.

[B26-behavsci-16-01898] Kim S. W. (2020). Meta-analysis of parental involvement and achievement in East Asian countries. Education and Urban Society.

[B27-behavsci-16-01898] Kim S. W., Hill N. E. (2015). Including fathers in the picture: A meta-analysis of parental involvement and students’ academic achievement. Journal of Educational Psychology.

[B28-behavsci-16-01898] Lakens D. (2017). Equivalence tests: A practical primer for t tests, correlations, and meta-analyses. Social Psychological and Personality Science.

[B29-behavsci-16-01898] Li J., Liu X., Zhu D., Jiang H. (2024). Effects of parent involvement in homework on students’ negative emotions in Chinese students: Moderating role of parent-child communication and mediating role of family responsibility. Behavioral Sciences.

[B30-behavsci-16-01898] Li Y., Hu T., Ge T., Auden E. (2019). The relationship between home-based parental involvement, parental educational expectation and academic performance of middle school students in mainland China: A mediation analysis of cognitive ability. International Journal of Educational Research.

[B31-behavsci-16-01898] Liu K. (2023). Middle school students’ mental unwellness and academic performance in China: The effects of parental involvement. PLoS ONE.

[B32-behavsci-16-01898] Liu S., Wang X., Ying J., Shi J., Wu X. (2023). Emotional involvement matters, too: Associations among parental involvement, time management and academic engagement vary with youth’s developmental phase. British Journal of Educational Psychology.

[B33-behavsci-16-01898] Liu Z., Hu J., Yang Q., Qiang Q., Hou X., Wang Z. (2025). Reciprocal relationship between parental educational involvement and depressive symptoms among adolescents: A four-year longitudinal study. Journal of Adolescent Health.

[B34-behavsci-16-01898] Moroni S., Dumont H., Trautwein U., Niggli A., Baeriswyl F. (2015). The need to distinguish between quantity and quality in research on parental involvement: The example of parental help with homework. The Journal of Educational Research.

[B35-behavsci-16-01898] Núñez J. C., Epstein J. L., Suárez N., Rosário P., Vallejo G., Valle A. (2017). How do student prior achievement and homework behaviors relate to perceived parental involvement in homework?. Frontiers in Psychology.

[B36-behavsci-16-01898] Patall E. A., Cooper H., Robinson J. C. (2008). Parent involvement in homework: A research synthesis. Review of Educational Research.

[B37-behavsci-16-01898] Pomerantz E. M., Moorman E. A., Litwack S. D. (2007). The how, whom, and why of parents’ involvement in children’s academic lives: More is not always better. Review of Educational Research.

[B38-behavsci-16-01898] Qin S., Jia S., Su S. (2025). Parent-child relationship and junior high academic performance: Differentiated impacts of fathers and mothers with self-efficacy as a mediator. Acta Psychologica.

[B39-behavsci-16-01898] Rosseel Y. (2012). lavaan: An R package for structural equation modeling. Journal of Statistical Software.

[B40-behavsci-16-01898] Ryan R. M., Deci E. L. (2000). Self-determination theory and the facilitation of intrinsic motivation, social development, and well-being. American Psychologist.

[B41-behavsci-16-01898] Sarkadi A., Kristiansson R., Oberklaid F., Bremberg S. (2008). Fathers’ involvement and children’s developmental outcomes: A systematic review of longitudinal studies. Acta Paediatrica.

[B42-behavsci-16-01898] Shi Z., Qu Y., Wang Q. (2024). Homework for learning and fun: Quality of mothers’ homework involvement and longitudinal implications for children’s academic and emotional functioning. Contemporary Educational Psychology.

[B43-behavsci-16-01898] Sijtsma K. (2009). On the use, the misuse, and the very limited usefulness of Cronbach’s alpha. Psychometrika.

[B44-behavsci-16-01898] Silinskas G., Niemi P., Lerkkanen M.-K., Nurmi J.-E. (2013). Children’s poor academic performance evokes parental homework assistance—But does it help?. International Journal of Behavioral Development.

[B45-behavsci-16-01898] Silinskas G., Viljaranta J., Poikkeus A.-M., Lerkkanen M.-K. (2022). Maternal homework approach and adolescents’ academic skills: The mediating role of task values. Frontiers in Education.

[B46-behavsci-16-01898] Solon G., Haider S. J., Wooldridge J. M. (2015). What are we weighting for?. Journal of Human Resources.

[B47-behavsci-16-01898] Tang X., Tang S., Ren Z., Wong D. F. K. (2020). Psychosocial risk factors associated with depressive symptoms among adolescents in secondary schools in mainland China: A systematic review and meta-analysis. Journal of Affective Disorders.

[B48-behavsci-16-01898] Trager B. M., Koning I. M., Rainosek L. M., Geurts S. M., van den Eijnden R. J. J. M., Vossen H. G. M. (2025). The longitudinal effects of parent–adolescent digital communication on depression and anxiety symptoms. Journal of Adolescent Health.

[B49-behavsci-16-01898] Vasquez A. C., Patall E. A., Fong C. J., Corrigan A. S., Pine L. (2016). Parent autonomy support, academic achievement, and psychosocial functioning: A meta-analysis of research. Educational Psychology Review.

[B50-behavsci-16-01898] Wang H., Zhan Z., Zhang Z. (2026). Parental involvement and children’s cognitive and non-cognitive development: Evidence from China. Frontiers in Psychology.

[B51-behavsci-16-01898] Wang M.-T., Sheikh-Khalil S. (2014). Does parental involvement matter for student achievement and mental health in high school?. Child Development.

[B52-behavsci-16-01898] Wang R., Li D., Zhang J., Song G., Liu Q., Tang X. (2022). The relationship between parent-adolescent communication and depressive symptoms: The roles of school life experience, learning difficulties and confidence in the future. Psychology Research and Behavior Management.

[B53-behavsci-16-01898] White I. R., Carlin J. B. (2010). Bias and efficiency of multiple imputation compared with complete-case analysis for missing covariate values. Statistics in Medicine.

[B54-behavsci-16-01898] Xu J., Du J., Wu S., Ripple H., Cosgriff A. (2018). Reciprocal effects among parental homework support, effort, and achievement? An empirical investigation. Frontiers in Psychology.

[B55-behavsci-16-01898] Xu J., Guo S., Feng Y., Ma Y., Zhang Y., Núñez J. C., Fan H. (2024). Parental homework involvement and students’ achievement: A three-level meta-analysis. Psicothema.

[B56-behavsci-16-01898] Xu Q., Wang J. (2019). Baba qu nar? Fuqin yu’er touru ji qi dui Zhongguo qingshaonian fazhan de yingxiang [Where is dad? Paternal involvement and adolescent development in China]. Shehui Fazhan Yanjiu [Journal of Social Development].

[B57-behavsci-16-01898] Yang Y. (2025). Reciprocal relationships between parental involvement and academic performance in early adolescence: A two-year longitudinal study in China. Journal of Youth and Adolescence.

[B58-behavsci-16-01898] Yap M. B. H., Morgan A. J., Cairns K., Jorm A. F., Hetrick S. E., Merry S. (2016). Parents in prevention: A meta-analysis of randomized controlled trials of parenting interventions to prevent internalizing problems in children from birth to age 18. Clinical Psychology Review.

[B59-behavsci-16-01898] Zapf H., Boettcher J., Haukeland Y., Orm S., Coslar S., Fjermestad K. (2024). A systematic review of the association between parent-child communication and adolescent mental health. JCPP Advances.

[B60-behavsci-16-01898] Zhang Q., Pan Y., Chen Y., Liu W., Wang L., Jean J. A. (2022). Effects of father-adolescent and mother-adolescent relationships on depressive symptoms among Chinese early adolescents. Applied Research in Quality of Life.

[B61-behavsci-16-01898] Zhang Q., Pan Y., Zhang L., Lu H. (2021a). Parent-adolescent communication and early adolescent depressive symptoms: The roles of gender and adolescents’ age. Frontiers in Psychology.

[B62-behavsci-16-01898] Zhang Q., Shek D. T. L., Pan Y. (2021b). Parent-child discrepancies in perceived parent-child communication and depressive symptoms in early adolescents in China. International Journal of Environmental Research and Public Health.

